# The Vip1 Inositol Polyphosphate Kinase Family Regulates Polarized Growth and Modulates the Microtubule Cytoskeleton in Fungi

**DOI:** 10.1371/journal.pgen.1004586

**Published:** 2014-09-25

**Authors:** Jennifer Pöhlmann, Carmen Risse, Constanze Seidel, Thomas Pohlmann, Visnja Jakopec, Eva Walla, Pascal Ramrath, Norio Takeshita, Sebastian Baumann, Michael Feldbrügge, Reinhard Fischer, Ursula Fleig

**Affiliations:** 1 Lehrstuhl für funktionelle Genomforschung der Mikroorganismen, Heinrich-Heine-Universität Düsseldorf, Düsseldorf, Germany; 2 Karlsruhe Institute of Technology (KIT) - South Campus, Institute for Applied Biosciences, Dept. of Microbiology, Karlsruhe, Germany; 3 Institut für Mikrobiologie, Heinrich-Heine-Universität Düsseldorf, Düsseldorf, Germany; 4 University of Tsukuba, Faculty of Life and Environmental Sciences, Ibaraki, Tsukuba, Japan; Duke University Medical Center, United States of America

## Abstract

Microtubules (MTs) are pivotal for numerous eukaryotic processes ranging from cellular morphogenesis, chromosome segregation to intracellular transport. Execution of these tasks requires intricate regulation of MT dynamics. Here, we identify a new regulator of the *Schizosaccharomyces pombe* MT cytoskeleton: Asp1, a member of the highly conserved Vip1 inositol polyphosphate kinase family. Inositol pyrophosphates generated by Asp1 modulate MT dynamic parameters independent of the central +TIP EB1 and in a dose-dependent and cellular-context-dependent manner. Importantly, our analysis of the *in vitro* kinase activities of various *S. pombe* Asp1 variants demonstrated that the C-terminal phosphatase-like domain of the dual domain Vip1 protein negatively affects the inositol pyrophosphate output of the N-terminal kinase domain. These data suggest that the former domain has phosphatase activity. Remarkably, Vip1 regulation of the MT cytoskeleton is a conserved feature, as Vip1-like proteins of the filamentous ascomycete *Aspergillus nidulans* and the distantly related pathogenic basidiomycete *Ustilago maydis* also affect the MT cytoskeleton in these organisms. Consistent with the role of interphase MTs in growth zone selection/maintenance, all 3 fungal systems show aspects of aberrant cell morphogenesis. Thus, for the first time we have identified a conserved biological process for inositol pyrophosphates.

## Author Summary

Fungi are an extremely successful and diverse group of organisms ranging from the small single-celled yeasts to the indefinitely growing filamentous fungi. Polarized growth, where growth is restricted to defined regions, leads to the specific cell shape of yeast cells, as well as the very long hyphae of filamentous fungi. Fungal polar growth is controlled by an internal regulatory circuit of which the microtubule cytoskeleton comprises the transport road for numerous cargos needed for polarized growth. However, the microtubule cytoskeleton is not static, but a dynamic structure, which is modulated by microtubule-associated proteins and the interaction with other cellular structures. Our present analysis has identified a new regulator of the microtubule cytoskeleton in the fission yeast *S. pombe*: a member of the highly conserved Vip1 inositol polyphosphate kinase family. Vip1 proteins have a dual domain structure consisting of an N-terminal kinase domain which synthesizes inositol pyrophosphates and a C-terminal domain, which we show to negatively regulate the kinase output. Our results suggest that modulation of microtubule dynamics is correlated to Vip1 kinase activity. Importantly, polarized growth and microtubule dynamics were also modulated by Vip1 family members in *A. nidulans* and *U. maydis* thus uncovering a conserved biological role for inositol pyrophosphates.

## Introduction

Cell polarization can be viewed as the generation and upkeep of a defined cellular organization. The readout of cell polarization in fungal systems is polarized growth resulting in a specific cell shape and size. This ranges from the 14 µm long cylindrical *Schizosaccharomyces pombe* fission yeast cell, which maintains its shape by restricting growth zones in a cell cycle dependent manner to the extremely polarized growth of filamentous fungi such as *Aspergillus nidulans* where hyphal extension can occur in a continuous and infinite manner [1]–[3]. Fungi are capable of morphological transitions in response to external signals and this represents an important virulence trait of pathogenic fungi such as the corn smut fungus *Ustilago maydis*. Here, the transition from a non-pathogenic haploid yeast-like form to a dikaryotic filament is required for the fungus to enter the host tissue [4]. Such an alteration in growth form is also present in non-pathogenic model yeasts such as *S. cerevisiae* and *S. pombe* where it acts as a foraging response [5]–[7]. Polarized growth in fungi depends on the interplay between the MT and actin cytoskeletons and in some systems septins [8]. In *S. pombe*, where growth occurs at the cell tips which contain oscillating Cdc42, actin cables are used for the transport of growth vesicles. On the other hand, MT plus-end dependent transport of the landmark complex Tea1-4 via the kinesin Tea2 is required for marking potential zones of growth [1], [9]–[13]. Correct delivery of Tea1-4 requires alignment of antiparallel interphase MTs along the long axis of the fission yeast cell. The dynamic MT plus-ends are oriented and polymerize towards the cell end; upon contact with the tip MT dynamics are modified, the landmark complex unloaded and anchored at the cell tip [14]–[16]. MT dynamics are regulated mainly by the diverse group of proteins at the MT plus-end. Here, the central component is the conserved EB1 family, which is essential for plus-end association of numerous +TIPs [17]. Interestingly, the Tea1-4 complex is also present in filamentous fungi where a recent publication has uncovered additional functions namely regulating MT dynamics and MT guidance at the hyphal tip. Loss of the *S. pombe* Tea1 homologue TeaA in *A. nidulans* results in an inability to maintain the direction of growth and thus results in meander-like growing hyphae [3], [18]. TeaA present at the hyphal tip is responsible for focusing of MTs at a single point and the regulation of MT plus-end dynamics via negative modulation of the XMAP214 family member AlpA [19]. If this negative regulatory function on MT dynamics is a common feature of Tea1-like proteins remains to be determined but the MT phenotype of *S. pombe tea1*Δ (deletion) cells supports such a scenario [14].

Although core mechanisms of growth zone definition and maintenance are conserved in fungi, the consistently growing hyphae of filamentous fungi require a much more sophisticated system of MT-based transport than is necessary for yeast cell growth [3], [20]–[27]. For example in *U. maydis* the MT cytoskeleton is required for long distance endosomal transport via plus- and minus-end -directed motor proteins such as kinesin and dynein, respectively [24], [28]–[32]. This transport process has been shown to be crucial for efficient secretion [33], [34]. Important molecular cargos of these endosomes are septins, mRNAs and ribosomes [35]–[37]. Interestingly, local translation of septin mRNA on shuttling endosomes loads these membranous carriers with newly synthesized septin protein for transport towards the hyphal tip [35].

In this work we describe a new core element of fungal growth zone selection and MT cytoskeleton regulation: the conserved Vip1 family which synthesizes diphospho-myo-inositol polyphosphates (inositol pyrophosphates). These high energy molecules are mainly made from inositol hexakiphosphate (IP_6_) and are generated by two classes of enzymes: IP6Ks and the PPIP5Ks (called Vip1 family throughout this work) (recently reviewed in: [38]–[41]. The Vip1 family, which was discovered in *S. pombe* and *S. cerevisiae*, was shown to have enzymatic activity by using an *S. cerevisiae* strain where the genes coding for the IP6K Kcs1 and the nudix hydrolase Ddp1 had been deleted [42], [43]. In humans two homologues exist named PPIP5K1 and PPIP5K2 [44], [45]. All members of this enzyme class have a dual domain structure consisting of an N-terminal “rimK”/ATP-grasp superfamily domain which phosphorylates position 1 on the fully phosphorylated inositol ring and a C-terminal domain with homology to histidine-acid-phosphatases [44]–[47]. The function of the latter domain remains a matter of debate. Key histidine residues are conserved in this domain, but unusually Vip1-like proteins do not have an aspartate residue next to the second histidine [45]. Furthermore detailed analysis of the human Vip1 phosphatase-like domain demonstrated that this domain is catalytically inactive [48]. However phenotypic analysis of *S. pombe* strains expressing Asp1 variants (fission yeast Vip1 member) with mutations of conserved C-terminal domain histidine residues suggested that these residues are required for negative regulation of the kinase activity [7]. In addition, truncated *S. cerevisiae* and human Vip1 variants which only contained the N-terminal kinase domain generated more inositol pyrophosphates than the full-length versions pointing to a kinase antagonizing activity of the C-terminal domain [45], [49]. Now, we provide evidence that Vip1-like proteins harbor two enzymatic activities.

Inositol pyrophosphates regulate cellular processes by two different modes of action: (i) modulation of protein function by reversible binding of these high energy molecules and (ii) protein pyrophosphorylation [50], [51]. An example for the first type of regulation is the Akt kinase which is involved in insulin signaling. Here, specific inositol pyrophosphates were shown to bind to the Akt PH domain thus blocking activation of this kinase [52]. An example for the second type of action is the regulation of the antiviral response via activation of the interferon transcription factor IRF3. In a cell free system IRF3 was phosphorylated by specific inositol pyrophosphates and this process required the transfer of the β-phosphate of the pyrophosphate group [49].

The cellular processes regulated by inositol pyrophosphates are wide-ranging and diverse. These include the phosphate availability response in *S. cerevisiae*, the chemotactic response in *Dictyostelium*, the antiviral response and insulin signaling in mammals and the dimorphic switch in *S. pombe* [7], [49], [50], [52], [53]. We have now extended our analysis of Vip1 biological function and have found that inositol pyrophosphates have a conserved role in fungal morphogenesis.

## Results

### The Vip1 family member Asp1 generates IP_7_ *in vitro*

**Figure 1:**
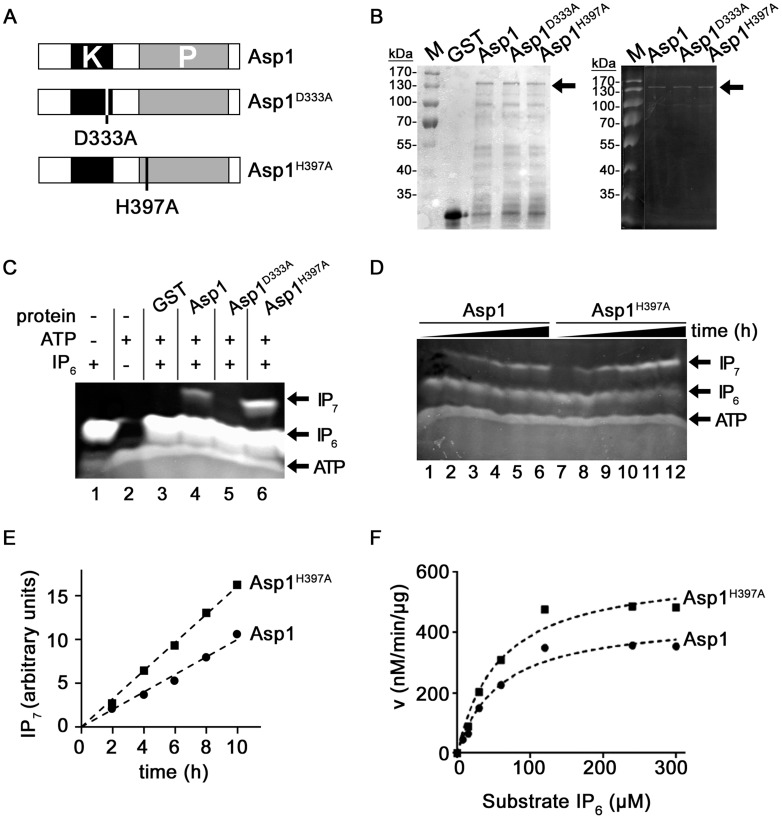
Generation of IP_7_ by Asp1 variants. (A) Diagrammatic representation of the Asp1 variants used. (B) Western blot analysis using a GST antibody of GST-tagged wild-type Asp1, GST-Asp1^D333A^ and GST-Asp1^H397A^. Proteins were purified from *E.coli*, quantified (Coomassie stained gel is shown in left panel) and equal amounts loaded on a 10% PAGE. The tagged Asp1 variants (arrow) run at the expected size of 139 kDa. (C) Generation of IP_7_ by GST-Asp1 variants. 4 µg of the indicated proteins were used in an ATP-dependent enzymatic reaction (16 hrs) and the resulting inositol pyrophosphates were resolved on a 35.5% PAGE and stained with Toluidine Blue [54]. −, component not present; +, component present. (D) Time-dependent (0–10 hrs in 2 hr. steps) generation of IP_7_ by Asp1 and Asp1^H397A^. Assay conditions were as in 1C. (E) Quantification and diagrammatic representation of the IP_7_ bands obtained in the assay shown in (D). (F) Determination of K_m_ and V_max_ values. 2 µg GST-Asp1 and GST-Asp1^H397A^ were incubated with varying amounts of substrate for 6 hrs. The IP_7_ was detected as in 1C, and quantified using an IP_6_ calibration curve (Materials and Methods). v, reaction rate. V_max_ Asp1: 450.5 nM/min/µg; V_max_ Asp1^H397A^: 611.2 nM/min/µg.

We had previously generated *S. pombe* strains that expressed the endogenous Asp1 variants Asp1^D333A^ and Asp1^H397A^ [7]. The former Asp1 variant has a single amino acid change at position 333, a key catalytic residue for kinase activity, while H397 is a highly conserved histidine residue of the C-terminal acid phosphatase-like domain (Figure 1A) [43]. Phenotypic analysis of these Asp1 variant expressing strains suggested that Asp1^D333A^ and Asp1^H397A^ have an altered enzymatic activity compared to the wild-type Asp1 protein [7]. We therefore assayed the ability of Asp1^D333A^ and Asp1^H397A^ to generate inositol pyrophosphates.

As it had not been demonstrated previously that the *S. pombe* Asp1 protein could generate inositol pyrophosphates, we first tested with an *in vitro* assay if this was the case. Asp1 was expressed in bacteria as a glutathione-S-transferase (GST) fusion protein and purified. Using a recently published method that allows analysis of inositol pyrophosphates by PAGE, we found that purified GST-Asp1 generated inositol pyrophosphates (from now on called IP_7_) in an ATP-dependent manner using IP_6_ as a substrate (Figure S1A, right panel) [54]. This activity was dose-dependent, as the amount of IP_7_ generated increased with increasing amounts of protein used (Figure S1B). We next tested if the GST-tagged Asp1^D333A^ and Asp1^H397A^ proteins (Figure 1B) also generated IP_7_ and found that Asp1^D333A^ was unable to convert IP_6_ to IP_7_ (Figure 1C, lane 5). Interestingly, comparing equal amounts of protein, the Asp1^H397A^ variant generated more IP_7_ than the wild-type Asp1 protein (Figure 1C, lane 6 and 4, respectively). Analysis of IP_7_ production by Asp1 and Asp1^H397A^ proteins over a time period of 0 to 10 hrs revealed that IP_7_ production increased with time and that Asp1^H397A^ could produce up to 100% more IP_7_ than the wild-type Asp1 protein (Figure 1D, left panel, lanes 7–12 and 1–6, respectively; quantification shown in 1E). Similar results were obtained when comparing IP_7_ production of the wild-type Asp1 and the Asp1^1-364^ variant (contains only the kinase domain) (Figure S12). These data point to a negative role of the C-terminal acid phosphatase-like domain. To analyze this further we determined the K_m_ and V_max_ values for Asp1 and Asp1^H397A^ (Figure 1F). The K_m_ values for Asp1 and Asp1^H397A^ were 58.18 µM and 57.32 µM respectively implying a similar affinity for the substrate. However the V_max_ for Asp1^H397A^ was 36% higher than that of Asp1 (Figure 1F).

**Figure 2:**
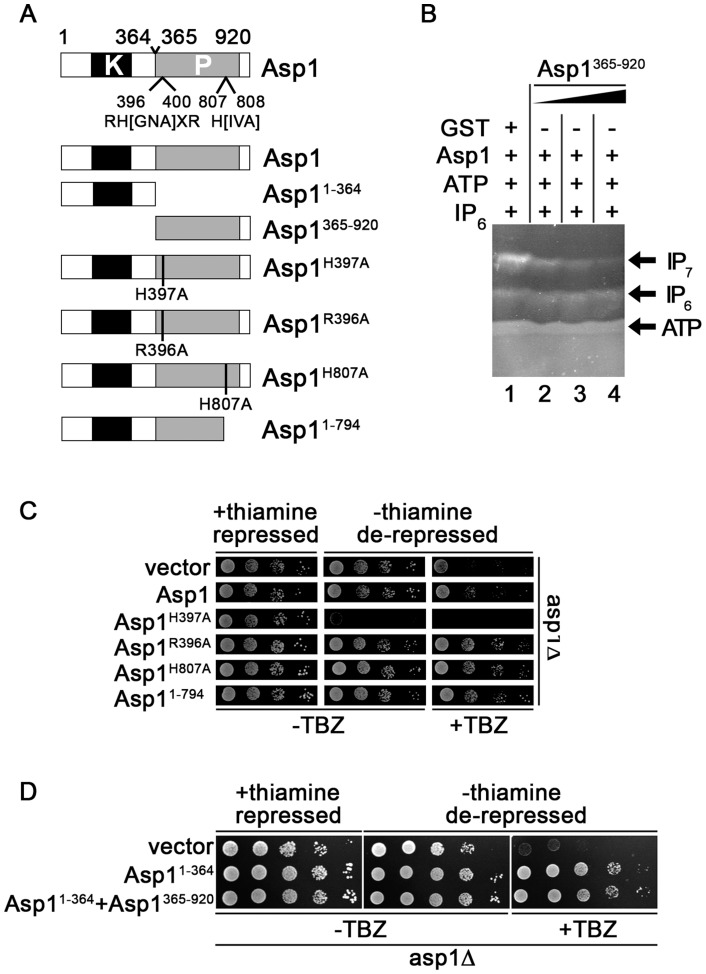
Function of the Asp1 phosphatase-like domain. (A) Diagrammatic representation of the Asp1 protein with the conserved phosphatase signature motif (motif: RH(GNA)XR-HD in Asp1 RHADR-HI)(top) and the Asp1 variants used. Top: (B) Generation of IP_7_ by GST-Asp1 with varying amounts (2,4,8 µg) of Asp1^365-920^. Enzymatic reaction was carried out as for 1C. −, component not present; +, component present. (C) Serial dilution patch tests (10^4^–10^1^ cells) of the *asp1*Δ (deletion) strain expressing the indicated *asp1* variants via the *nmt1^+^* promoter. This promoter is repressed in the presence of thiamine and de-repressed in its absence. Cells were grown for 6 days at 25°C on plasmid selective minimal medium without (−) or with (+) TBZ. (D) Serial dilution patch tests (10^5^–10^1^ cells) of the *asp1*Δ strain expressing either *asp1^1-364^*, *asp1^365-920^* or *asp1^1-364^* and *asp1^365-920^*. Cells were grown for 7 days at 25°C on plasmid selective minimal medium without (−) or with (+) TBZ.

To learn more about the negative impact of the Asp1 phosphatase-like domain on IP_7_ production, we (i) tested if addition of Asp1^365-920^ reduced the inositol pyrophosphate out-put in an Asp1 *in vitro* kinase assay and (ii) determined the *in vivo* read-out of Asp1 variants with mutations in conserved residues of the phosphatase-like domain (Figure 2A).

The presence of purified bacterially expressed GST-tagged Asp1^365-920^ in the IP_7_ *in vitro* assay together with full length Asp1 reduced the amount of IP_7_ in a dose-dependent manner (Figure 2B). IP_6_ amounts were unaffected by Asp1^365-920^ as shown by the incubation of only this Asp1 variant with IP_6_ in the *in vitro* assay (Figure S13). Thus, the negative effect was only seen for the IP_7_ output. We therefore propose that the Asp1 C-terminal phosphatase-like domain has phosphatase activity and its substrate is inositol pyrophosphate generated by the Asp1 N-terminal kinase domain (see discussion).

We had shown previously that the *asp1^H397A^* strain was more resistant to microtubule poisons such as thiabendazole (TBZ) while the *asp1^D333A^* strain was more sensitive to TBZ compared to the wild-type strain [7]. A deletion of *asp1^+^* (*asp1*Δ strain) also led to TBZ hypersensitivity (Figure S2A). Furthermore a strain where the wild-type *asp1^+^* had been replaced by the *asp1^D333A, H397A^* variant also showed the same increased TBZ sensitivity as the *asp1^D333A^* and *asp1*Δ strains (Figure S2A). These data strongly suggest that the TBZ resistance/sensitivity of these strains is solely dependent on the function of the Asp1 kinase. Absence of Asp1 kinase activity results in TBZ hypersensitivity (*asp1*Δ, *asp1^D333A^* and *asp1^D333A, H397A^* strains) while increased Asp1 kinase function (*asp1^H397A^* strain) results in TBZ resistance. The Asp1 C-terminal phosphatase-like-domain appears to modulate only the function of the Asp1 N-terminal kinase domain as the *asp1^D333A, H397A^* strain has the same TBZ phenotype as *asp1*Δ and *asp1^D333A^* strains (Figure S2A).

These results demonstrate that increased TBZ resistance can be used as an *in vivo* read-out for a non-functional Asp1 phosphatase domain. We expressed wild-type *asp1^+^* and the mutant versions *asp1^H397A^*, *asp1^R396A^*, *asp1^H807A^* and *asp1^1-794^* on a plasmid from the thiamine-repressible *nmt1^+^* promoter [55] in the *asp1*Δ strain. Western blot analysis revealed that expression of full length Asp1 variants was similar (Figure S14). Expression of these *asp1* variants except *asp1^H397A^* did not affect cell growth (Figure 2C, growth on thiamine (*nmt1^+^* promoter repressed) versus growth on thiamine-less (*nmt1^+^* promoter de-repressed) plates. Plasmid-borne high expression of *asp1^H397A^* is lethal as has been shown previously [43].

As shown in Figure 2C plasmid-borne expression of full length *asp1^+^* allowed partial growth of the *asp1*Δ strain on TBZ containing plates. However expression of Asp1^R396A^, Asp1^H807A^ and Asp1^1-794^ resulted in better growth of the *asp1*Δ strain on TBZ medium. We conclude that the conserved phosphatase signature motif is required for the function of the C-terminal domain.

To test if the Asp1 C-terminal domain is also able to regulate Asp1 kinase activity *in trans in vivo*, we constructed a plasmid, which expressed Asp1^1-364^ and Asp1^365-920^ from two separate *nmt1^+^* promoters on the same plasmid (Figure S3A). Expression of this plasmid in the *asp1*Δ strain resulted in a similar phenotype as expression of a plasmid expressing only Asp1^1-364^ (Figure 2D, protein levels shown in Figure S3B–C). Thus in this *in vivo* situation, it appears that both Asp1 domains need to be on the same molecule for the negative impact of the C-terminal domain to be exerted.

### Asp1 affects interphase MT organization

Our *in vitro* kinase assay demonstrated that the Asp1^D333A^ variant has no enzymatic activity, while that of Asp1^H397A^ is higher than that of the wild-type Asp1 protein. Thus it is very likely that the resistance/sensitivity to microtubule poisons is a result of different intracellular inositol pyrophosphate levels.

We had previously identified a truncated Asp1 variant (Asp1^1-794^) as a multi-copy suppressor of the TBZ-hypersensitivity of a *mal3* mutant strain [7]. Mal3 is the fission yeast member of the EB1 family of MT associated proteins [56]. We therefore determined if Asp1 function modulated the MT cytoskeleton by analyzing the interphase MT cytoskeleton of the various *asp1* strains via expression of GFP-α-tubulin (using the endogenous *nmt81*::*gfp-atb2^+^* construct) [57]. *asp1* variant strains with or without the presence of GFP-α-tubulin had a similar growth phenotype (Figure S4A).

**Figure 3:**
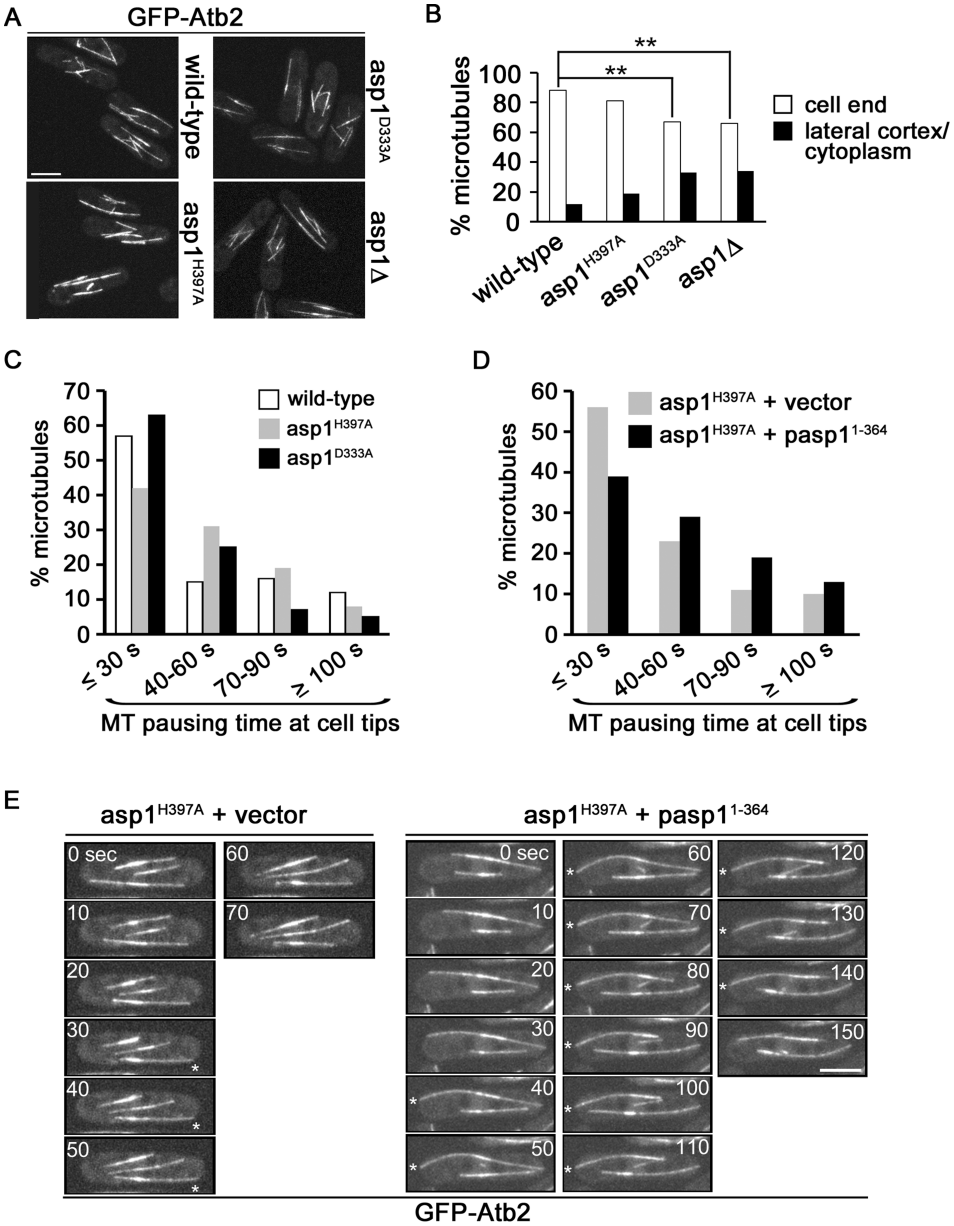
Asp1 kinase function affects MT organization. (A) Live cell images of the indicated strains expressing *nmt81::gfp-atb2^+^*. The same imaging and image-processing conditions were used for all strains. Bar, 5 µm. (B) Percentage of MTs depolymerising at a cell end or at the lateral cortex/in the cytoplasm. Wild-type: n = 102, *asp1^H397A^*: n = 218, *asp1^D333A^*: n = 166, *asp1*Δ: n = 131. ** P<0.005 for *asp1^D333A^* or *asp1*Δ compared to wild-type as determined using χ^2^-test. (C) MT pausing time (sec) at cell ends in the indicated strains. Overall MT pausing time of these strains is shown in table 1. We arbitrarily defined the 4 categories to show the variability within this system. Wild-type: n = 100, *asp1^H397A^*: n = 67, *asp1^D333A^*: n = 75. (D) MT pausing time (sec) at cell ends in the *asp1^H397A^* strain transformed with a vector control or expressing pasp1^1-364^. Overall MT pausing time of these strains is shown in table 1. Cells were grown in plasmid-selective minimal medium. *asp1^H397A^*+vector: n = 105, *asp1^H397A^*+p*asp1^1-364^*: n = 110. pasp1^1-364^ denotes plasmid-borne expression of Asp1^1-364^ via the *nmt1^+^* promoter under promoter de-repressing conditions. (E) Live cell images of the *nmt81*::*gfp*-*atb2* ^+^ expressing *asp1^H397A^* strain transformed with the vector control or expressing pasp1^1-364^. Images shown are 10 sec intervals. Asterisks (*) denote MTs touching the cell end. Bar, 5 µm.

In *S. pombe*, interphase MTs are polymerized in the vicinity of the nucleus, align along the long axis of the cell and grow with their plus-ends towards the cell end, where they pause prior to de-polymerization [58]. The organization of interphase MTs of the *gfp-atb2 asp1^H397A^* strain was comparable to the wild-type strain while those of the fainter fluorescent *gfp-atb2 asp1^D333A^* and *gfp-atb2 asp1*Δ MTs appeared to be more disorganized (Figure 3A). In particular, the number of interphase MTs that were not oriented along the long axis of the cell was increased in *asp1^D333A^* (Figure S15) and *asp1*Δ cells although this was not statistically significant. However the number of interphase MTs that depolymerized at the lateral cortex/cytoplasm and not at the cell tip was increased significantly in *asp1^D333A^* and *asp1*Δ cells compared to wild-type cells (Figure 3B). An example is shown for an *asp1^D333A^* MT that touched the lateral cortex and became depolymerized instead of being deflected as seen for such MTs in *asp1^+^* cells (Figure S4B).

### Inositol pyrophosphates regulate interphase MT dynamics

Measurement of MT parameters in the 3 *asp1* variant strains revealed that MT dynamics were altered. *asp1^D333A^* cells showed an increased MT growth rate while the rate of MT shrinkage was decreased in *asp1^H397A^* cells (Table 1). Interphase MTs of *asp1^D333A^* cells had an increased number of catastrophe events while those of *asp1^H397A^* cells were reduced compared to wild-type cells (Table 1). The average MT length for both *asp1* mutant strains was increased compared to the MTs of the wild-type strain. Thus, all measured MT parameters were affected by intracellular inositol pyrophosphate levels. *asp1^D333A^* MTs are more dynamic, whereas *asp1^H397A^* MTs have the opposite phenotype.

**Table 1:** Interphase MT dynamics in *asp1* variant strains.

strain	growth (nm/sec)	rate of shrinkage (nm/sec)	growth before catastrophe (sec)	length (µm)	pausing at tips (sec)
wild-type	56.8±26.5 (n = 90)	154.8±66.5 (n = 72)	81.2±32.3 (n = 100)	6.3±1.2 (n = 100)	47.3±38.0 (n = 100)
asp1^D333A^	66.7±27.2* **↑** (n = 90)	145.6±67.5 (n = 45)	68.3±29.4* **↓** (n = 126)	6.9±1.1* **↑** (n = 86)	34.5±29.1* **↓** (n = 75)
asp1^H397A^	58.2±30.9 (n = 90)	126.7±50.2* **↓** (n = 92)	99.0±39.2* **↑** (n = 155)	7.3±1.4* **↑** (n = 100)	50.7±35.7 (n = 67)

Parameters of MT dynamics were measured for the indicated strains expressing *nmt81::gfp-atb2^+^*. Cells were grown in non-selective minimal medium. n, number of MTs measured; asterisks denote significance between wild-type and mutant; ↓ or ↑, parameter is decreased or increased compared to wild-type. growth: * p<0.01 for *asp1^D333A^* vs. wild-type (t-test). Rate of shrinkage: * p<0.005 for *asp1^H397A^* vs. wild-type (Welch-test). Growth before catastrophe: * p<0.005 for *asp1^D333A^* vs. wild-type (t-test), * p<0.0005 for *asp1^H397A^* vs. wild-type (t-test). Length: * p<0.0005 for *asp1^D333A^* vs. wild-type (t-test), * p<0.0005 for *asp1^H397A^* vs. wild-type (Welch-test). Pausing at tips: * p<0.01 for *asp1^D333A^* vs. wild-type (Welch-test).

Interestingly, we found that the residence time of the MT plus-end at the cell tip was dependent on the *asp1* variant expressed in the cell. Measurement of the time that a MT stays at the cell tip showed that the residence time of a MT plus-end at the cell tip is variable. Nevertheless, when we compared this MT parameter for wild-type and *asp1^D333A^* cells we found that the latter MTs had on average a significantly reduced pausing time at the cell tip before depolymerization (Table 1). For example, only 12% of *asp1^D333A^* MTs but 28% of wild-type MTs paused at the cell tip for more than 60 seconds (Figure 3C). In contrast, the residence time of *asp1^H397A^* MT plus-ends at the cell tip appeared to be increased compared to wild-type MTs but this was not statistically significant (Table 1). We therefore increased Asp1 generated inositol pyrophosphate levels even further by plasmid-borne expression of the Asp1 variant Asp1^1-364^ (kinase only) in the *asp1^H397A^* strain. Under these conditions the average MT pausing time was increased by 30% in cells expressing Asp1^1-364^ (*asp1^H397A^* strain plus vector: 42.2±36.8 seconds; n = 105; *asp1^H397A^* strain plus pasp1^1-364^: 53.6±38.4 seconds; n = 110; p<0.025 (t-test)). A detailed depiction of MT pausing time in this assay is shown in Figure 3D and an example of the increased MT residence at the cell tip is shown in Figure 3E.

Thus, inositol pyrophosphate levels appear to regulate the residence time of a MT plus-end at the cell tip. Increasing the levels of Asp1 generated inositol pyrophosphates increases pausing at the tip prior to a catastrophe event, while lowering the amount of Asp1 generated inositol pyrophosphates has the opposite effect.

### Asp1 regulated MT dynamics occur independently of the +TIP protein Mal3

**Figure 4:**
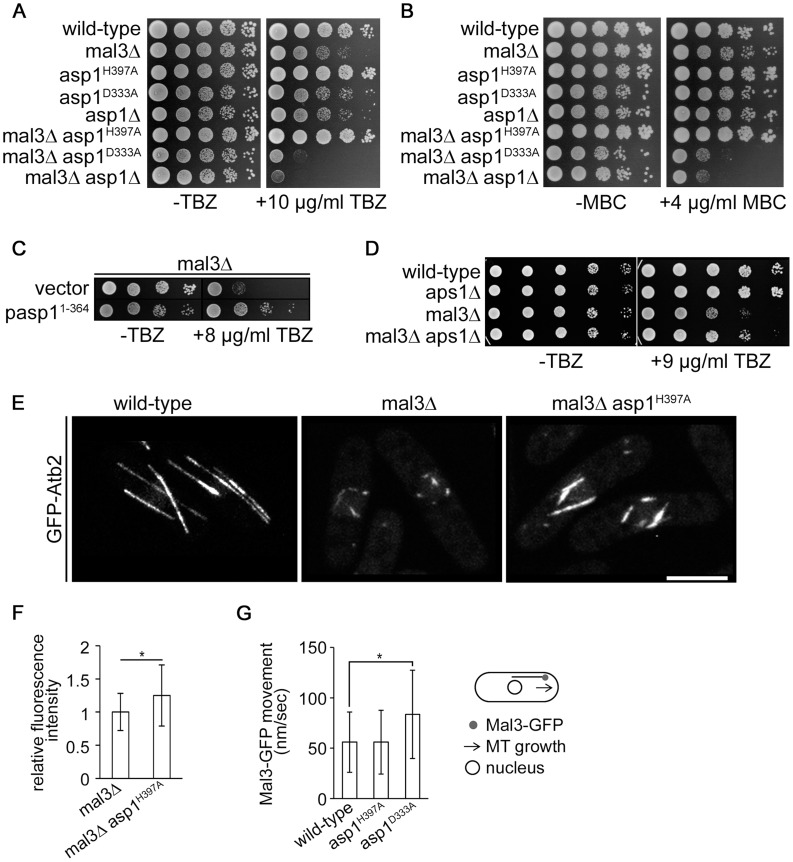
Asp1 MT regulation functions independently of Mal3. (A) Serial dilution patch test (10^4^–10^1^ cells) of the indicated strains grown for 5 days at 25°C on YE5S without (−) or with (+) TBZ. (B) Serial dilution patch test (10^4^–10^1^ cells) of the indicated strains grown for 5 days at 25°C on YE5S without (−) or with (+) MBC. (C) Serial dilution patch test of the *mal3*Δ transformants grown under plasmid selective conditions at 25°C for 5 or 9 days without (−) or with (+) TBZ, respectively. (D) Serial dilution patch tests (10^5^–10^1^ cells) of the indicated strains grown at 25°C on MM without (−) or with (+) TBZ. (E) Photomicrographs of living wild-type, *mal3*Δ and *mal3*Δ *asp1^H397A^* cells grown at 30°C expressing *nmt81::gfp-atb2* ^+^. Bar, 5 µm. (F) MT relative fluorescent intensity (*mal3*Δ strain, 1+/−0.28, n = 26; *mal3*Δ *asp1^H397A^* strain, 1.25+/−0.46, n = 16; arbitrary units; *, *P*<0.05 as determined using Welch-Test). (G) Movement of outmost outbound Mal3-GFP comets (see diagram). Speed of comets (nm/sec): wild-type, 56±30, n = 93; *asp1^H397A^*, 56±31.6, n = 69; *asp1^D333A^*, 83.7±43,8, n = 75. * p<0.0005 for *asp1^D333A^* versus wild-type (Welch-test).

Proteins associated with MT plus-ends play a leading role in regulating MT dynamics [59]. Of particular importance is the EB1 family, which is central to the association of other +TIPs with the MT plus-end. To determine if Asp1 affects MT dynamics via the EB1 family member Mal3, double mutant strains between *mal3*Δ (*mal3* deletion) and the *asp1* alleles *asp1^H397A^*, *asp1^D333A^* and *asp1*Δ were constructed. The *asp1^H397A^ mal3*Δ strain showed a reduced TBZ sensitivity compared to the single mutant *mal3*Δ strain, demonstrating that increased Asp1 kinase function rescues the *mal3*Δ mutant TBZ phenotype (Figure 4A). Loss of Asp1 kinase activity increased the TBZ hypersensitivity of *mal3*Δ strains as shown for the *asp1^D333A^ mal3*Δ and *asp1Δ mal3*Δ strains (Figure 4A). Similar results were obtained when these strains grew on medium containing the MT drug methyl-benzimidazol-2-yl-carbamate (MBC) (Figure 4B).

We next assayed if plasmid borne overexpression of the Asp1 variant Asp1^1-364^ (Asp1 kinase domain only), rescued the *mal3*Δ TBZ hypersensitivity, and found this to be the case (Figure 4C). Furthermore increasing intracellular IP_7_ levels by other means than *asp1* ^+^ manipulation, namely by using a strain where the ORF coding for the nudix hydrolase Aps1 was deleted (*aps1*Δ), also decreased the TBZ hypersensitivity of the *mal3*Δ strain (Figure 4D). Nudix hydrolases degrade inositol pyrophosphates and disruption of the nudix hydrolase encoding gene increases the intracellular concentration of inositol pyrophosphates 3-fold [60], [61].

As Mal3 stabilizes MTs, *mal3*Δ cells do not have a normal interphase MT-cytoskeleton, where MTs are aligned in antiparallel bundles along the cell axis to the cell ends. Instead such interphase cells have very short MT stubs present around the nucleus as MT catastrophe events are increased (compare wild-type MTs to *mal3*Δ MTs) (Figure 4E) [56], [62], [63].

This short interphase MT phenotype was rescued partially in the *mal3*Δ *asp1^H397A^* strain (Figure 4E).We determined MT parameters in the *mal3*Δ and *mal3*Δ *asp1^H397A^* strains and observed no difference in the number of MTs/cell (Figure S16). An analysis of the interphase MTs of a *mal3*Δ *asp1^D333A^* strain was not possible as interphase MTs of this strain were extremely short and unstable.

Interestingly, MT length, MT growth time and the relative MT intensity were all increased significantly in the double mutant *mal3*Δ *asp1^H397A^* strain compared to the single mutant *mal3*Δ strain (Table 2 and Figure 4F). MTs grew longer before a catastrophe event in the *mal3*Δ *asp1^H397A^* strain compared to the *mal3*Δ strain and MT length was increased in the former compared to the latter strain (Table 2). Furthermore the relative MT fluorescence intensity was increased 1.25 fold in the *mal3*Δ *asp1^H397A^* strain compared to *mal3*Δ strain (Figure 4F). Thus MT dynamics regulation by inositol pyrophosphates does not require the EB1 protein.

**Table 2:** Interphase MT dynamics in *mal3Δ* variant strains.

strain	growth (nm/sec)	length (µm)
mal3Δ	37±23 (n = 90)	2.1±0.3 (n = 54)
mal3Δ asp1^H397A^	54±35* **↑** (n = 91)	2.7±0.6* **↑** (n = 53)

Parameters of MT dynamics were measured for the indicated strains expressing *nmt81::gfp-atb2^+^*. Cells were grown in non-selective minimal medium. n, number of MTs measured; asterisks denote significance between *mal3Δ* and *mal3Δ asp1^H397A^*; ↑, parameter is increased compared to *mal3Δ*. Growth: * p<0.0005 for *mal3Δ asp1^H397A^* vs. *mal3Δ* (t-test). Length: * p<0.0005 for *mal3Δ asp1^H397A^* vs. *mal3Δ* (t-test).

Next, we analyzed Mal3-GFP particle movement in the various *asp1* strains. The EB1 family decorates MTs and forms the comet-shaped structures at the MT plus-end characteristic of plus-end tracking proteins [59], [63]. The Mal3-GFP distribution on MTs was similar in all *asp1* strains and was as described [63]. We determined the speed of the outmost outbound Mal3-GFP comets moving towards the cell end. As shown in Figure 4G movement of such Mal3-GFP particles in the wild-type and *asp1^H397A^* strain was similar, while *asp1^D333A^* Mal3-GFP comets were faster. The speed of movement of outmost outbound Mal3-GFP was directly correlated to the MT growth rate of the particular *asp1* strain (Table 1 and Figure 4G). We also assayed movement of the kinesin Tea2-GFP in the 3 *asp1* variant strains and found that the speed of Tea2-GFP signals at the end of MTs was comparable to Mal3-GFP comets (Figure S17).

### Asp1 kinase function is required for growth zone selection in *S. pombe*

Interphase MTs in *S. pombe* control proper polarized growth by delivering the Tea1-4 landmark protein complex to potential sites of growth at the cell tip [10], [12], [64], [65]. Consequently, an aberrant interphase MT cytoskeleton can result in an altered positioning of the growth zones and in cells with a branched or bent morphology.

In wild-type fission yeast cells growth at a specific cell end is cell cycle controlled. After cytokinesis, cells will first grow in a monopolar manner selecting the old end (the end present before the previous cell division) as the first growth zone. The attainment of a critical cell size and completion of S-phase allow a switch to bipolar growth (NETO transition) at both cell ends in the G2 cell cycle phase [66], [67]. We had shown previously that Asp1 kinase function is essential for NETO, as 84% of *asp1^D333A^* cells grow exclusively monopolar on an agar surface using the old end as the site of growth [7]. However, the cylindrical cell shape was maintained in most *asp1^D333A^* cells demonstrating that the growth zone was still at the cell end. Abnormal growth zone positioning i.e. the selection of a growth zone not at the cell tip was observed in less than 5% of *asp1^D333A^* cells [7].

**Figure 5:**
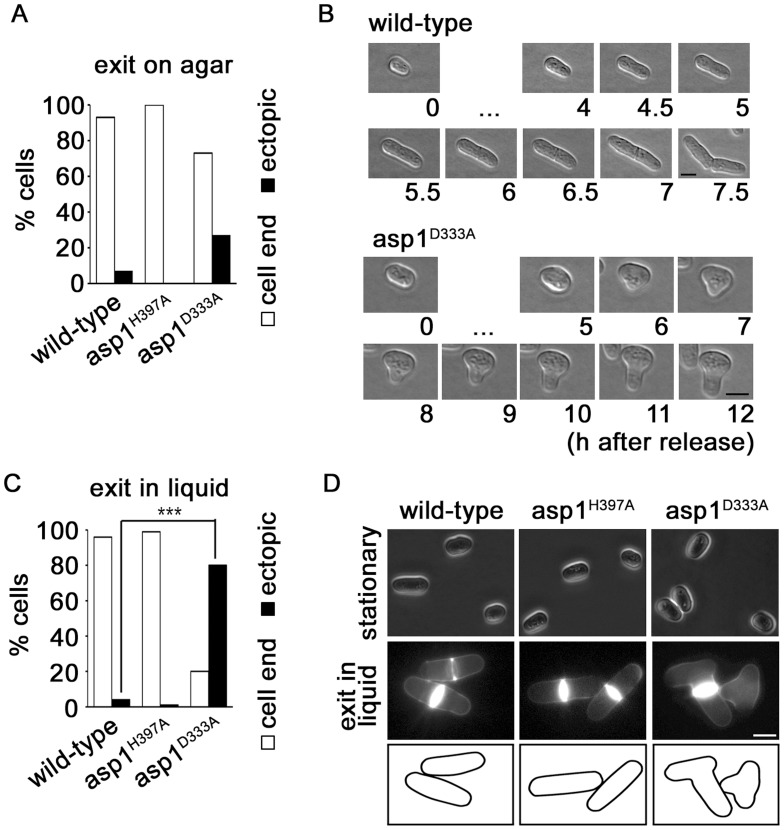
Asp1 is required for growth zone selection. (A) The indicated strains were released from stationary phase by streaking cells on solid YE5S at 25°C and analyzing the cell shape microscopically. Wild-type strain, n = 16; asp1^H397A^, n = 19; asp1^D333A^ strain, n = 15. (B) Photomicrograph of a wild-type and *asp1^D333A^* cell that exits stationary phase on solid agar. Bars, 5 µm. […] denotes no change in cell morphology at these time points. (C) The indicated strains were released from stationary phase by inoculating an aliquot with YE5S liquid medium. Phenotype was scored after 7 hrs at 25°C. Wild-type strain, n = 142; *asp1^H397A^*, n = 110; *asp1^D333A^* strain, n = 122 ***p<0.001 *asp1^D333A^* compared to wild-type as determined using χ^2^-test. (D) Photomicrographs of cells in stationary phase (top panels) and after release into YE5S liquid medium (bottom panels). Cells were stained with Calcofluor white. Bar, 5 m.

Next we asked, if proper polarized growth could also be re-established in *asp1* mutants that were re-entering the vegetative cell cycle after nutrient starvation. Re-entry of cells into the cell cycle from G0 requires a *de novo* definition of the growth zones. [13], [16]. We thus examined the morphology of *asp1^+^* and *asp1* mutant cells after exit from stationary phase: on agar 93% of *asp1^+^*, 100% of *asp1^H397A^* but only 73% of *asp1^D333A^* cells grew as normal cylindrically shaped cells (Figure 5A). The remaining 27% of growing cells had an abnormal morphology, indicating that proper polarized growth was not re-established (Figure 5A–B). Incubation of stationary *asp1^D333A^* cells into fresh liquid medium massively aggravated the ectopic growth phenotype: under these conditions 80% of the cells had an aberrant, branched or lemon-shaped appearance indicating that Asp1 kinase activity was required for polarized growth and growth zone selection under these circumstances (Figure 5C–D). We also determined if cells when exiting from G0 state on solid medium showed the monopolar to bipolar growth pattern of exponentially growing cells. However we found a wide variety of growth patterns even for the *asp1^+^* cells indicating that cells need to undergo a number of cell divisions before the normal growth pattern is stably re-established. It was thus not possible to determine if *asp1^D333A^* cells deviate from the norm.

### *A. nidulans* Vip1-like protein is required for polarized growth

As the Vip1 family is conserved from yeast to man, we determined if Vip1 members also played a role in cell morphogenesis in other organisms. We therefore analyzed the function of Asp1-homologues in the filamentous ascomycete *Aspergillus nidulans* and the dimorphic basidiomycete *Ustilago maydis*. In both fungi, the importance of the MT cytoskeleton for fungal growth has been investigated extensively [18], [21]–[26], [31], [68], [69]. We decided to generate and characterize strains where the genes coding for the Asp1-homologues had been deleted as we have shown for *S. pombe* that the *asp1*Δ strain behaved identical to the *asp1^D333A^* strain under all conditions tested (Figure S2A–B; [7]).

**Figure 6:**
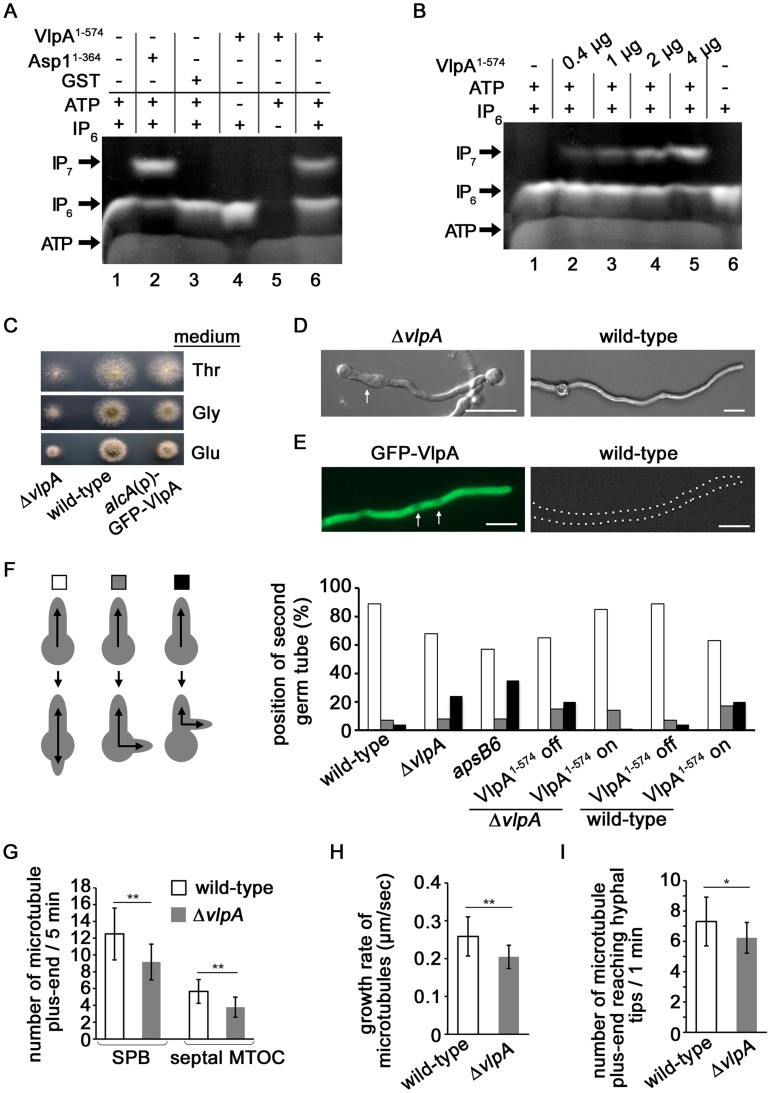
Function of Vip1-like protein *A. nidulans* VlpA. (A) VlpA has kinase activity. 2 µg GST-VlpA^1-574^ or GST-Asp1^1-364^ (kinase domain only) were used in an enzymatic reaction as described [54]. −, component not present in assay; +, component present in assay. (B) Correlation between GST-VlpA^1-574^ protein amount/assay and the amount of IP_7_ generated. (C) Colonies of the *vlpA*-deletion strain Δ*vlpA* (left), wild-type (middle) and a strain in which GFP-VlpA was expressed under the control of *alcA* promoter (right). The strains were grown on minimal medium agar with threonine (upper), glycerol (middle) or glucose (bottom) for 2 days at 37°C. (D) The Δ*vlpA* strain (SCoS94, left) and wild-type strain (right) were grown in minimal medium with glucose overnight at 28°C. Some hyphae showed swelling in the Δ*vlpA* strain (arrow). Bars, 10 µm. (E) Growth conditions of the GFP-VlpA strain (Scos176) as in (D). GFP-VlpA expressed under native promoter localized predominantly in the cytoplasm with weak nuclear staining (arrows). The wild-type strain photographed under the same condition is shown on the right. Bars, 10 µm. (F) Left panel: position of the second germtube or branch: second germtube opposite first germtube (white), random position (grey) or second hypha branching out of the first hypha (black). Right panel: quantification of the number of germlings with a second germtube in the indicated strains. Spores were grown in minimal medium with glucose overnight. To induce expression of VlpA^1-574^, minimal medium with glycerol was used. N germlings/strain = 100. (G) Number of MT plus-ends appearing from the SPB or septal MTOC during 5 minutes in the wild-type (SSK92, white) and the *vlpA*-deletion strain (SDO2, grey). At SPB, wild-type: n = 20, Δ*vlpA*: n = 12, **p<0.01 as determined using t-test. At MTOC, wild-type: n = 10, Δ*vlpA*: n = 9, **p<0.01 as determined using t-test. (H) Growth rate of MT in the wild-type (SSK92) and the Δ*vlpA* strain (SDO2, grey). N = 10 cells for each of the strains, **p<0.01 as determined using t-test. (I) Number of MT plus-ends reaching hyphal tips during 1 minute in the wild-type (SSK92) and Δ*vlpA* strain (SDO2). N = 13 cells for each of the strains, *p<0.05 as determined using t-test.

The *A. nidulans* Asp1 orthologue AN5797.2 has the characteristic Vip1 family dual domain structure (Figure S5) and was named *vlpA* (Vip1-like protein). To test if VlpA generates inositol pyrophosphates, bacterially expressed and purified GST-VlpA^1-574^, which contains the putative kinase domain was used in the *in vitro* kinase assay [54]. VlpA^1-574^ generated IP_7_ in an ATP dependent manner using IP_6_ as a substrate (Figure 6A, lanes 6, 4 and 5, respectively). This activity increased with increasing amounts of VlpA^1-574^ (Figure 6B).

We next deleted the endogenous *vlpA* gene and found that the *vlpA*-deletion strain (Δ*vlpA*) showed a growth delay and smaller colonies than the wild-type strain (approximate 50% diameter of colony on glucose medium) (Figure 6C). The majority of hyphae in the Δ*vlpA* strain displayed a normal morphology however swelling of hyphae was observed in some instances (Figure 6D, left). This phenotype could be caused by mis-positioning of the growth zone.

We constructed a strain expressing N-terminally GFP-tagged VlpA fusion protein under the control of the inducible *alcA* promoter instead of native VlpA. Under repressed conditions with glucose as carbon source, the strain exhibited a growth delay (Figure 6C, bottom right most panel). Under de-repressed conditions with glycerol or induced conditions with threonine, the slow growth phenotype was alleviated implying that GFP-VlpA can complement the growth defect of the *vlpA* deletion. We constructed a strain expressing GFP-VlpA under the native promoter and found that GFP-VlpA fluorescence was observed predominantly in the cytoplasm (Figure 6E, left).

Interestingly, the *A. nidulans* VlpA is needed for correct growth zone selection as it is required for the correct positioning of the second germtube. Once the first hypha reaches a determinate length, a second germ tube appears on the spore after the first septum at the base of the first hypha was formed [70]. This second germination site normally lies opposite of the first hypha (Figure 6F). In *A. nidulans*, MTs are formed from spindle pole bodies (SPB) and from septum-associated MT-organizing centers (septal-MTOCs) [71], [72]. MTs emanating from the septum of the first hypha grow towards the first germtube as well as into the direction of the spore. The MTs from the septa towards the spore are required for the positioning of the second germtube [70]. In the *vlpA* deletion strain, 24% of the spores did not produce a second germtube from the spore but produced a second hypha by branching out of the first hypha situated between septum and spore (Figure 6F). This aberrant phenotype was rescued by expressing a VlpA variant GFP-VlpA^1-574^ (contains the kinase domain) from the *alcA* promoter in the *vlpA* deletion strain (Figure 6F). Interestingly, expression of this VlpA variant in the wild-type strain altered growth zone selection (Figure 6F). These results demonstrate that (i) VlpA kinase activity is required for growth zone selection and (ii) physiological levels of VlpA kinase are required for proper growth zone selection. Thus, the Vip1-like proteins from *A. nidulans* and *S. pombe* are both required for growth zone selection.

### *A. nidulans* VlpA modulates the MT cytoskeleton

A comparable phenotype of aberrant growth zone selection had been observed previously for the *apsB6* mutant (Figure 6F) [70]. The *apsB* gene was identified by mutant screening. Anucleate primary sterigmata (aps) mutants are partially blocked in conidiation due to failure of the organized migration of nuclei into the conidiophore metulae. The mutants also show irregular distribution of nuclei in vegetative hyphae [73]. ApsB is a MTOC component that interacts with gamma-tubulin [74]. The *apsB6* mutant shows an altered MT organization as it forms fewer MTs out of SPBs, compared to the wild-type and substantially fewer MTs from septa [72]. We therefore analyzed such parameters in the *vlpA*-deletion strain.

GFP tagged KipA, which is a kinesin localizing at growing MT plus-end, was used as plus-end marker to determine MT parameters [71]. Comparing wild-type to the *vlpA*-deletion strain during a five minute time period, we observed a reduction of newly emanating GFP-KipA signals in the *vlpA*-deletion strain at SPBs (27%) and at septal-MTOC (33%) (Figure 6G, Figure S6, Movie S1 and S2). The growth rate of the MT plus-ends was slightly reduced in the *vlpA*-deletion strain (21%) (Figure 6H). Pausing of MT plus-ends at hyphal tips was analyzed by using GFP-α-tubulin. Since the pausing time at hyphal tips was too short to determine if differences existed between the wild-type and the *vlpA* deletion strains, we scored the number of MT plus-ends reaching hyphal tips during a 1 minute time period. We counted fewer MT plus-ends in the *vlpA* deletion strain compared to the wild-type strain indicating that MT dynamics at the hyphal tip was altered in the absence of VlpA (Figure 6I).

### The Asp1-like protein UmAsp1 is important for proliferation and polar growth in *U. maydis*

**Figure 7:**
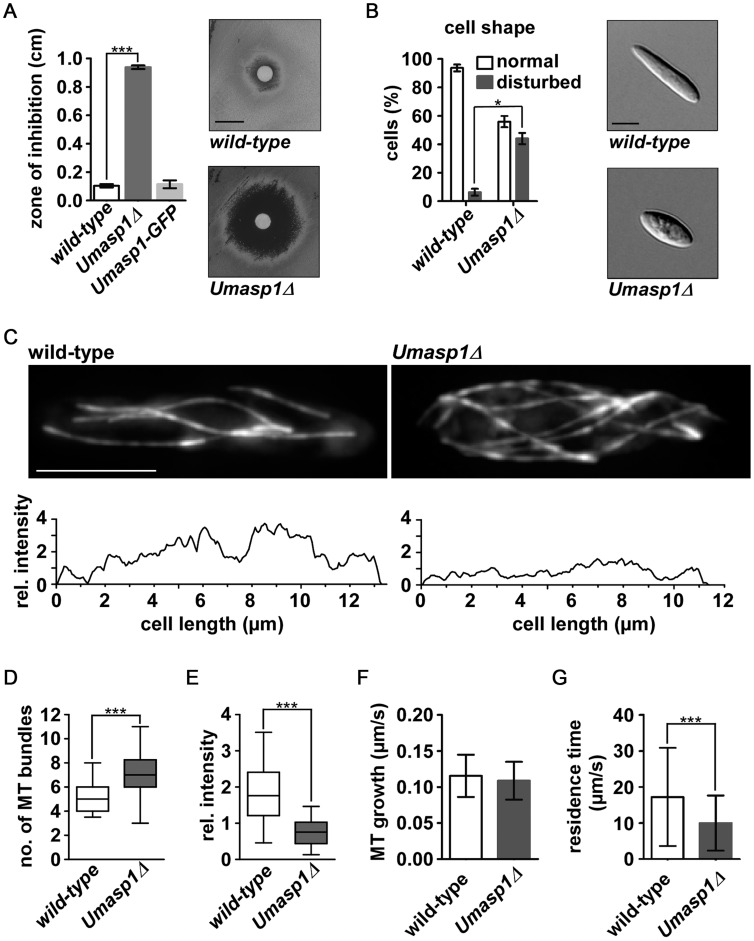
Loss of *Umasp1* causes aberrant morphology of and altered MT organization in *U. maydis* yeast cells. (A) Diagrammatic representation of growth inhibition test. The radius of growth zone inhibition was determined for the indicated strains on plates with 10 µl TBZ (concentration: 10 mg/ml) at the centre (experiments, n = 3. Error bars show SEM. ***, p<0.001; t-test). Representative examples are shown on the right and in Figure S7C (size bar, 1 cm). (B) Percentage of cells with disturbed cell shape. Bars show the mean of three independent experiments with n>100 cells (error bar shows SEM. *p<0.03, t-test). Representative examples are shown on the right (size bar, 5 µm). (C) Top: Deconvolved fluorescence photomicrographs depicting MT morphology (via expression of GFP-Tub1 (GFP fused to α-tubulin)) of wild-type and *Umasp1*Δ cells (size bar, 5 µm). Note that due to deconvolution fluorescence for the *Umasp1*Δ cell appears brighter. Bottom: Corresponding intensity profile showing longitudinal maximum intensity of background subtracted raw images. (D and E) Whisker diagrams showing the number of wild-type and *Umasp1*Δ MT bundles (D) and their relative intensity (E). Whiskers indicate 90%/10% percentiles (n>49 cells in (D) and n = 10 cells in (E); *** p<0.001 Mann-Whitney test for (D) and (E)). (F) MT growth parameters. Growth of MTs was determined by analyzing the comet-like movement of Pep1-GFP (error bars indicate standard deviation). Only MTs that grew >2 µm were analyzed (n = 225 and 125 for wild-type and *Umasp1*Δ, respectively). (G) The residence time of dynamic MTs was determined in GFP-Tub1 strains. n = 79 and 96 for wild-type and *UMasp1*Δ respectively. Error bar indicates standard deviation (unpaired t-test, *** p<0.001).

Finally, we studied the function of an Asp1 homologue in a distantly related fungus, the basidiomycete *U. maydis*. Sequence comparison revealed a protein designated UmAsp1(um06407 in MUMDB; MIPS *Ustilago maydis* database [75], with 922 amino acids and 49% sequence identity to *S. pombe* Asp1 over its entire length (Figure S5). To study its function we generated deletion strains in laboratory strain AB33. This strain is a derivative of wild-type strain FB2 that contains an active bW2/bE1 heterodimeric transcription factor under control of the nitrate-inducible promoter P_nar1_. Thereby, b-dependent filamentation can be elicited by changing the nitrogen source in the medium [76]. We observed that a corresponding deletion strain of *Umasp1*Δ exhibited reduced proliferation during yeast-like growth in comparison to wild-type (Figure S7). Assaying TBZ sensitivity revealed that *Umasp1*Δ strains were hypersensitive to this MT inhibitor (Figure 7A; Figure S7B–C). For microscopic analysis we compared wild-type and *Umasp1*Δ strains expressing GFP-Tub1 (GFP fused to α-tubulin). The *Umasp1*Δ strain showed an increased number of cells that were clearly different from the cigar-shaped wild-type cells. Cells exhibited an increased diameter in the central region and/or were rounded-up at the poles (Figure 7B; Figure S8). Such cells were classified as having a disturbed shape and quantification revealed that about 40% of *Umasp1*Δ cells had an abnormal cell morphology (Figure 7B). Analysis of the MT cytoskeleton showed specific deviations from wild-type MTs. In wild-type cells 4 to 5 microtubular bundles are observed that are facing with their plus ends towards the poles [23], [25], [77]. We observed that the MT organization was altered in *Umasp1*Δ cells: a conservative quantification scoring only cells with drastic changes revealed that in comparison to the wild-type MT organization was altered (Figure S9). The most profound differences observed were (i) *Umasp1*Δ cells with large buds exhibited depolymerized MTs (Figure S9B, bottom panels); a phenotype rarely observed for wild-type cells. (ii) *Umasp1*Δ cells mostly with no bud or a small bud (early G2 phase) [23] had significantly more MT bundles. Instead of the 4 to 5 bundles present in wild-type cells, we observed 6 to 8 (Figure 7C–D; Movies S3 and S4). The fluorescence intensity of the GFP-Tub1 signal was drastically reduced in these bundles (Figure 7C, E) suggesting that loss of UmAsp1 results in an increased number of MT bundles with fewer MTs within such a bundle.

Studying the subset of intact MTs indicated that MT growth rate, which was analyzed by determining the velocity of the GFP-tagged *U. maydis* EB1 protein Pep1 [77], was not significantly different compared to wild-type (Figure 7F), but the residence time of MTs pausing at the cell end was significantly reduced (Figure 7G). In summary, UmAsp1 is needed for correct morphology and MT organization during proliferation of yeast-like cells.

**Figure 8:**
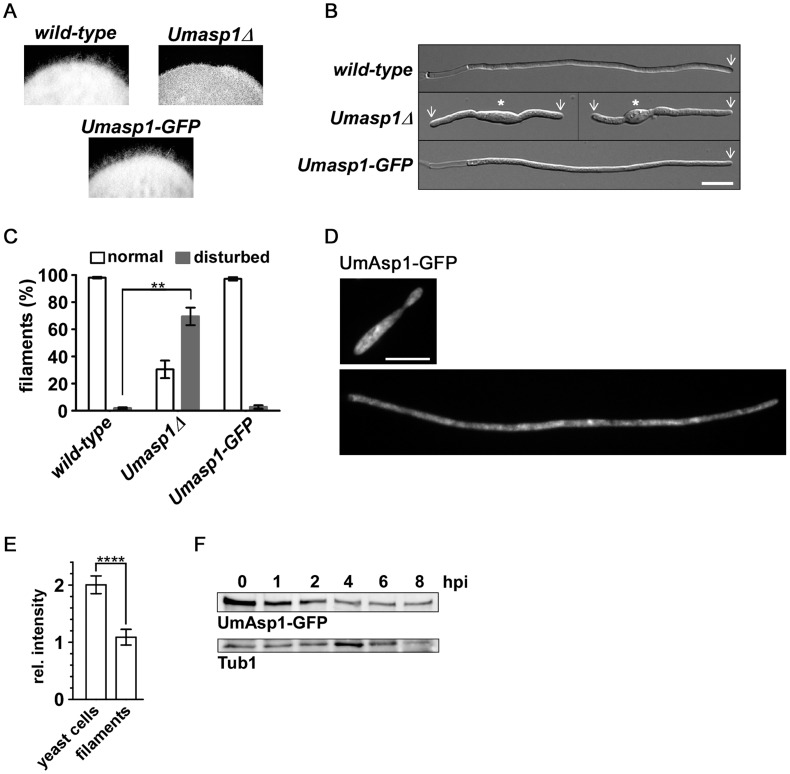
Loss of UmAsp1 causes defects in filamentous growth. (A) Edges of colonies of the indicated AB33 derived strains grown on charcoal plates. Aerial hyphae are emanating from the colony. (B) Photomicrographs (DIC) of the indicated strains grown for 8 hrs under filament inducing conditions. Wild-type and UmAsp1-GFP filaments form characteristic empty sections at the basal pole. White arrow: growth zone; white star: yeast cell (bar, 10 µm). (C) Bar diagram showing percentage of filaments exhibiting normal or disturbed growth. Bars show the mean of three independent experiments with n>100 cells (error bar, SEM; ** p = 0.0108). (D) Examples of UmAsp1-GFP yeast cells and filaments (8 hours post induction) (bar, 10 µm). (E) Bar chart showing mean average fluorescence intensity of UmAsp1-GFP in yeast and hyphae (yeast, n = 10 cells and hyphae, n = 7 cells; see Figure S11 for example photomicrographs). Error bars indicate standard deviation (*** p<0.001 unpaired t-test). (F) Western blot analysis of protein extracts of strain AB33 Umasp1-GFP after induction (0–8 hrs) of filamentous growth. Tub1 served as a loading control (hpi, hours post induction).

To investigate the function of UmAsp1 during hyphal growth, AB33 filamentation was induced on plates and in liquid medium. Wild-type forms a fuzzy colony indicative for efficient hyphal growth (Figure 8A, top, left panel). This was disturbed in *Umasp1*Δ strains (Figure 8A, top, right panel). Filaments were shorter, often bipolar and the amount of abnormal filaments was clearly increased in *Umasp1*Δ strains (Figure 8B–C, Figure S10). Thus, as in hyphae of *A. nidulans*, UmAsp1 is important for filamentous growth.

To study the subcellular localization we generated strains expressing UmAsp1-GFP (C-terminal fusion to GFP). The resulting strain was phenotypically indistinguishable from wild-type (Figures 7A, 8A–C) demonstrating that the fusion protein is fully functional. Studying the subcellular localization in yeast or hyphal cells did not reveal any pronounced subcellular accumulation of the protein as has been shown for other Vip1-like proteins (Figure 8D). However, UmAsp1-GFP fluorescence was reduced in hyphae, suggesting that the protein amount decreases after filament induction (Figure 8E, Figure S11). Indeed, western blot analysis demonstrated that UmAsp1-GFP protein amounts decreased over time (Figure 8F). Thus, UmAsp1 protein amounts decrease and hence presumably intracellular inositol pyrophosphate levels appear to be down-regulated during the switch to hyphal growth.

## Discussion

In this work we have defined the function of the C-terminal domain of the Vip1 family member Asp1 from *S. pombe* and have identified a new role for inositol pyrophosphates in fungal polarized growth and the modulation of MTs. In all three fungal model systems analyzed transport-based processes along the MT cytoskeleton are essential for proper polarized growth. However the long hyphal compartments of the filamentous fungi require a more sophisticated system of localized delivery [3], [18], [24]. Thus although Vip1-like proteins play a role in polarized growth in *S. pombe*, *A. nidulans* and *U. maydis* their specific roles are not expected to be identical.

### Function of the Asp1 C-terminal histidine acid phosphatase domain

All Vip1 family members have a dual domain structure consisting of an N-terminal kinase domain and a C-terminal histidine acid phosphatase-like domain. Generation of inositol pyrophosphates has been shown for the budding yeast and human Vip1 family members [43]–[45], [48]. In this work we have extended the analysis to two further fungal Vip1-like proteins: the *S. pombe* Asp1 and the *A. nidulans* VlpA. Both proteins generated inositol pyrophosphates *in vitro*. The use of Asp1 and VlpA N-terminal- only-variants mapped the kinase activity to the N-terminal part of the respective protein.

**Figure 9:**
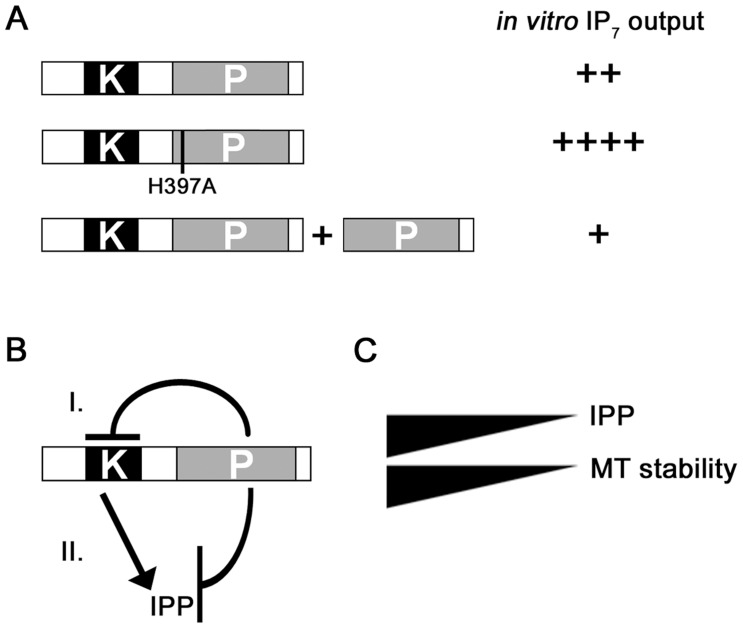
Model for the regulation of Asp1 kinase function by the C-terminal phosphatase domain. (A) *In vitro* IP_7_ output of wild-type Asp1 (top), Asp1^H397A^ (middle) and Asp1 plus Asp1^365-920^ (phosphatase domain only). ++++ - +, high to low IP_7_ output. (B) Two possible modes of action are shown. (I) The phosphatase domain could modulate the function of the kinase domain directly leading to reduced inositol pyrophosphate generation. (II) The phosphatase domain has enzymatic activity using the inositol pyrophosphate generated by the kinase domain as a substrate. IPP = inositol pyrophosphate. (C) MT stability correlates directly with intracellular inositol pyrophosphate levels.

The precise function of the C-terminal phosphatase-like domain of Vip1-like proteins has been elusive. The histidine acid phosphatase signature motif is in principle present in Vip1-like proteins but the conserved “HD” motif has been replaced by H(I,V,A) [45]. A recent publication has shown that the phosphatase-like domains of the human Vip1 members are catalytically inactive. Instead the authors show that this domain plays a role in inositol lipid binding [48]. On the other hand, a comparison of the amounts of inositol pyrophosphates generated by human and the *S. cerevisiae* full-length Vip1 proteins versus N-terminal kinase-domain-only-variants, showed that the latter variants exhibited more specific activity [43], [45]. This implied a negative impact of the phosphatase-like domain on inositol pyrophosphate production. However it was unclear, if this effect was due to the large size differences between the full length and the kinase-domain-only-variants [45]. In this paper we demonstrate that the phosphatase-like domain has a regulatory function: (i) the Asp1^H397A^ variant generated significantly more inositol pyrophosphates *in vitro* than the equally sized wild-type Asp1 protein (Figure 9A). The K_m_ values for these two proteins were similar, but V_max_ for the mutant Asp1 variant Asp1 ^H397A^ was higher. (ii) Addition of the phosphatase-only variant Asp1^365-920^ to an Asp1 protein containing *in vitro* kinase assay massively reduced the IP_7_ output (Figure 9A). However the presence of a mutated phosphatase variant, Asp1^365-920 H397A^ in the assay did not have this effect (Figure S18). Thus our results suggest that the C-terminal phosphatase-like domain of Asp1 has enzymatic activity and its substrates are the inositol pyrophosphates produced by the N-terminal kinase domain of the protein (Figure 9B, model II). However as we have not formally proven that the C-terminal domain has phosphatase activity other modes of regulation are possible as shown in model I (Figure 9B).

We have shown previously that specific extrinsic signals appear to up-regulate Asp1 kinase activity via the cAMP PKA pathway [7]. We speculate that such an up-regulation might occur by modification and result in down-regulation of the Asp1 C-terminal domain function. Such a scenario could also be envisaged for other external signal induced processes regulated by Vip1 family members, such as the antiviral response [49].

### Inositol pyrophosphate signaling is an important modulator of fungal growth

The present work has defined a new role for inositol pyrophosphates generated by the Vip1 family: the modulation of fungal growth and the MT cytoskeleton. In *S. pombe*, interphase MT organization and MT dynamics were strongly altered in the *asp1* mutant strains. In *A. nidulans* MT arrays from the SPB and the septal MTOC were affected in the *vlpA* deletion strain while in *U. maydis* loss of the Vip1-like protein resulted in increased TBZ sensitivity and an increase of cells with aberrant MT organization.

How then do inositol pyrophosphates modulate the MT cytoskeleton? In all systems tested to date and shown for *U. maydis* and *A. nidulans* in this work, Vip1 proteins are predominantly cytoplasmic without a specific subcellular localization [42], [48]. However as inositol pyrophosphates appear to modulate processes by binding to proteins or by pyrophosphorylation of proteins, direct association of Vip1 proteins with their targets might not be necessary. Our analysis in *S. pombe* demonstrated that in the absence of the +TIPs EB1 family member Mal3 the MT cytoskeleton can still be modulated by inositol pyrophosphates. Furthermore MT localization of EB1 proteins appeared unaffected in the *S. pombe asp1^D333A^* and the *Umasp1*Δ strain. As the EB1 protein family is at the center of the +TIP network of MT plus-ends and required for the recruitment of the majority of +TIPs [17], [59], we reason that such MT proteins are unlikely targets of inositol pyrophosphates. We have started to search for MT relevant inositol pyrophosphate targets by expressing either *asp1^1-364^* (kinase domain only) or *asp1^365-920^* (phosphatase domain only) in various *S. pombe* mutants with an altered MT cytoskeleton. Our rationale is that the mutant phenotype of a strain with a deletion of a direct Vip1 target should not be affected by varying inositol pyrophosphate levels. We found that inositol pyrophosphates show a “genetic interaction” with the MT plus-end components that can associate with MTs independently of EB1 (our unpublished observations). However other MT structures might also be modulated by inositol pyrophosphates: MTs emanating from SPBs and septal MTOCs are reduced in the *A. nidulans vlpA*-deletion strain as has been shown for the *apsB* mutant strain [72]. ApsB is a conserved MTOC associated protein that interacts with γ-tubulin [74].

Of particular interest is the observed direct correlation between intracellular inositol pyrophosphate levels and the time that *S. pombe* MT plus-ends stay at the cell tip before a catastrophe event. Components of a fungal growth zone can regulate MT plus-end dynamics as has been shown for *A. nidulans* Tea1 family member TeaA, which negatively regulates the activity of the XMAP215 protein AlpA [19]. Thus it is feasible that Asp1 enzymatic activity regulates MT dynamics at the cell tip. Although immunofluorescence analysis of *S. pombe* Asp1-GFP did not show a specific cytoplasmic localization [42], localization of the human Vip1 member PPIP5K1 was slightly enhanced at the plasma membrane [48]. Plasma membrane targeting of PPIP5K1 in NIH3T3 cells was increased dramatically following PtdIns3 kinase activation [48].

### Inositol pyrophosphates and growth zone selection

In fission yeast the switch from mono- to bipolar growth (NETO) is a complicated process that is regulated by a number of interwoven processes [10], [12]. These range from the correct positioning of landmark proteins by the MT cytoskeleton to the successful completion of S-phase and cytokinesis [64]–[66], [78]. We have shown previously that *asp1^D333A^* cells are able to correctly initiate growth at the old end after cytokinesis but cannot undergo NETO [7]. Correct selection of the first growth zone was also observed for the positioning of the first germtube of spores of an *A. nidulans vlpA*-deletion strain. However, similar to the *S. pombe* NETO event the positioning of the second growth zone (second germtube) was aberrant. Interestingly, plasmid-borne expression of *A. nidulans* VlpA^1-574^ (kinase domain) in a wild-type background also led to an alteration in the positioning of the second germ tube. We presume that VlpA^1-574^ expression increases intracellular inositol pyrophosphate levels and thus propose that fine-tuning of inositol pyrophosphate levels is required for the correct positioning of the second germ tube in *A. nidulans*.

In accordance with this hypothesis we observed that hyphal growth was also disturbed in *U. maydis*. Loss of UmAsp1 caused reduced and aberrant filamentous growth, including an increase of bipolar filaments. This is reminiscent of strains treated with MT-inhibitors or carrying mutations in MT-dependent motors such as kinesin-3 type Kin3, dynein Dyn1/2 or missing the RNA-binding protein Rrm4 involved in endosomal mRNA transport [35]–[37].

Interestingly, UmAsp1 levels decreased after the switch to hyphal growth indicating that alternative growth forms require a modulation of intracellular inositol pyrophosphate levels. Noteworthy, these filaments are arrested in the G2 cell cycle [79] suggesting a connection to cell cycle control.

A change in inositol pyrophosphate levels also regulates the environmentally controlled switch to an alternative growth form of *S. pombe* namely pseudohyphal invasive growth [7]. Here, Asp1 generated inositol pyrophosphates were essential for the switch to occur and increasing intracellular levels of these high energy molecules increased the cellular response. A similar scenario has been described recently for the regulation of the antiviral response by human Vip1 generated inositol pyrophosphates [49]. Ectopic expression of human Vip1 family members strongly increased the interferon response. Thus, modulation of the kinase activity of Vip1-like proteins might be a general mechanism of eukaryotic cells to react to extrinsic signals.

## Materials and Methods

### *In vitro* enzymatic activity of Vip1-like proteins

PCR-generated DNA fragments containing the *S. pombe asp1^+^, asp1^D333A^*, *asp1^H397A^ asp1^364-920^* ORFs, the *S. cerevisiae VIP^1-535^* and the *A. nidulans vlpA^1-574^* were cloned into *E. coli* expression vector pKM36 (a gift from Dr. K. Mölleken, Heinrich-Heine-Universität, Düsseldorf, Germany) to generate GST-tagged proteins. These proteins were expressed and purified from *E.coli* strain Rosetta (DEB) according to protocol (Sigma Aldrich). Protein concentration was determined using Bradford. Defined quantities of the Vip1-like proteins were used in an enzymatic reaction followed by PAGE analysis [54]. Intensity of IP_7_ bands was determined with ImageJ 1.44 (NIH). Determination of K_m_ and V_max_: Enzymatic reactions with 2 µg of protein were carried out for 6 hrs using 0–300 µM IP_6_ substrate. The amount of IP_7_ generated per reaction was determined by quantifying the relevant IP_7_ band and converting this number using an IP_6_ calibration curve. IP_6_ was obtained from Sigma-Aldrich. Michaelis-Menten enzyme kinetics were calculated with GraphPad Prism6 (GraphPad Software, Inc.).

### Strains and media

All strains used are listed in Table 3. *S. pombe* strains were grown and new strains were obtained as described [7]. *A. nidulans* was grown in supplemented minimal medium including 2% glucose, 2% glycerol or 2% threonine [80]. *A. nidulans* strain constructions were as described [81].To generate a N-terminal GFP fusion construct of VlpA a 900 bp fragment of *vlpA* (starting from ATG) was amplified from genomic *A. nidulans* DNA with appropriate primers. This *Asc*I-*Pac*I-digested PCR fragment was cloned into the corresponding sites of pCMB17apx (for N-terminal GFP fusion proteins of interest expressed under the control of *alcA* promoter, containing *Neurospora crassa pyr4* as a selective marker) [82], generating pCoS105. The 1.5-kb promoter of *vlpA* was amplified from genomic DNA with appropriate primers and cloned into the corresponding sites of pCoS105, generating pCoS228. They were transformed into wild-type strain TN02A3. To express VlpA variant GFP-VlpA^1-574^ (contains the kinase domain) from the *alcA* promoter, the fragment of *vlpA* was amplified from genomic *A. nidulans* DNA with appropriate primers. This *Asc*I-*Pac*I-digested PCR fragment was cloned into the corresponding sites of pCMB17-pyroA (*pyr-4* was replaced with *pyroA* in pCMB17apx), generating pCoS197, which was transformed into the wild-type strain TN02A3 and *vlpA*-deletion strain. Integration event was confirmed by PCR. *vlpA* was deleted via transformation of a deletion cassette (Program Project grant GM068087) into TN02A3 and the deletion confirmed by southern blotting. *U. maydis* strain constructions and growth of yeast like cells was performed according to published protocols [76]. Filamentous growth of AB33 and variants was induced by shifting 20 or 50 ml of exponentially growing cells (OD_600_ = 0.4–0.5) from complete medium (CM) to nitrate minimal medium each supplemented with 1% glucose. Cells were incubated at 28°C shaking at 200 rpm for 4 to 8 h prior to microscopy. For serial dilution patch tests, cells were pre-grown to OD_600_ = 0.5 before plating. For quantitative inhibition studies, cells were grown to OD_600_ = 0.5 and 300 µl were streaked out on a CM-plate. The filter paper present at the plate centre contained either 10 µl DMSO (solvent control) or 10 µl TBZ (10 mg/ml). After three days of growth at 28°C the radius of growth inhibition was measured.

**Table 3:** Strains used in this study.

*S. pombe*		
name	genotype	source
UFY1156	h^−^ asp1Δ::kan^R^ his3-D1 ade6-M216 leu1-32 ura4-D18	U. Fleig
UFY605	h^−^ his3-D1 ade6-M210 leu1-32 ura4-D18	K. Gould
UFY1579	h^+^ asp1^H397A^::kan^R^ his3-D1 ade6-M210 leu1-32 ura4-D18	U. Fleig
UFY1511	h^+^ asp1^D333A^::kan^R^ his3-D1 ade6-M210 leu1-32 ura4-D18	U. Fleig
UFY857	h^−^ kan^R^::nmt81::gfp-atb2^+^ leu1-32	T. Toda
UFY963	h^+^ kan^R^::nmt81::gfp-atb2^+^ ade6-M216 his3D1 leu1-32	This study
UFY1318	h^+^ kan^R^::nmt81::gfp-atb2^+^ ade6-M210 his3D1 leu1-32 ura4-D18	This study
UFY1763	h^+^ asp1^H397A^::kan^R^ kan^R^::nmt81::gfp-atb2^+^ leu1-32 ura4-D18	This study
UFY1529	h^+^ asp1^D333A^::kan^R^ kan^R^::nmt81::gfp-atb2^+^ leu1-32 ura4-D18	This study
UFY1407	h^+^ asp1Δ::kan^R^ kan^R^::nmt81::GFP-atb2^+^ leu1-32 his3-D1 ade6-M216	This study
UFY135	h^+^ mal3Δ::his3^+^ his3Δ ade6-M210 leu1-32 ura4-D18	U. Fleig
UFY561	h^+^ mal3Δ::ura4^+^ ade6-M210 his3Δ leu1-32 ura4-D18	U. Fleig
UFY1641	h^−^ asp1^H397A^::kan^R^ mal3Δ::his3^+^ his3^−^ ade6-M210 leu1-32 ura4-D18	U. Fleig
UFY1528	h^−^ asp1^D333A^::kan^R^ mal3Δ::his3^+^ his3^−^ ade6-M210 leu1-32 ura4-D18	This study
UFY1322	h^−^ asp1Δ::kan^R^ mal3Δ::his3^+^ his3^−^ ade6-M216 leu1-32 ura4-D18,	This study
UFY1729	h^−^ aps1Δ::his3^+^ his3-D1 ade6-M210 leu1-32 ura4-D18	U. Fleig
UFY2164	h^+^ aps1Δ::his3^+^ mal3Δ::ura4^+^ his3^−^ ade6-M210 leu1-32 ura4-D18	This study
UFY880	h^−^ mal3Δ::ura4^+^ kan^R^::nmt81::gfp-atb2^+^ his3Δ ade6-M210 leu1-32 ura4-D18	This study
UFY1762	h^−^ asp1^H397A^::kan^R^ mal3Δ::ura4^+^ kan^R^::nmt81::gfp-atb2^+^ his3^−^ ade6-M210 leu1-32 ura4-D18	This study
UFY596	h^−^ mal3-pk-GFP::ura4^+^ ade6-M210 his3D1 leu1-32 ura4-D18	H. Browning
UFY2015	h^−^ asp1^H397A^::kan^R^ mal3-pk-GFP::ura4^+^ ade6-M210 his3D1 leu1-32 ura4-D18	This study
UFY2014	h^+^ asp1^D333A^::kan^R^ mal3-pk-GFP::ura4^+^ ade6-M210 his3D1 leu1-32 ura4-D18	This study
UFY1582	h^−^ asp1^D333A H397A^::kan^R^ ade6-M210 leu1-32 ura4-D18 his3-D1	This study

*A. nidulans*		
name	genotype	source
TN02A3	*pyrG89*; *argB2*, Δ*nkuA*::*argB*; *pyroA4*	[88]
SDV25	*alcA*(p)::gfp::kipA:: *pyr-4*; *pyrG89*; *apsB6*	[72]
SSK92	*alcA*(p)::gfp::kipA:: *pyr-4*; *pyrG89*; *pyroA4*	[71]
SCoS90	*alcA*(p)::gfp::*vlpA* ^900bp^:: *pyr-4*; *pyrG89*; *argB2*, Δ*nkuA*::*argB*; *pyroA4*	This study
SCoS176	*vlpA*(p)::*gfp::vlpA* ^900bp^:: *pyr-4*; *pyrG89*; *argB2*, Δ*nkuA*::*argB*; *pyroA4*	This study
SCoS153	*alcA*(p)::*gfp::vlpA* ^1-574^:: *pyroA*; *pyrG89*; *argB2*, Δ*nkuA*::*argB*; *pyroA4*	This study
SCoS155	*alcA*(p)::*gfp::vlpA* ^1-574^:: *pyroA*; Δ*vlpA*:: *pyr-4*; *pyrG89*; *argB2*, Δ*nkuA*::*argB*; *pyroA4*	This study
SCoS94	Δ*vlpA*:: *pyr-4*; *pyrG89*; *argB2*, Δ*nkuA*::*argB*; *pyroA4*	This study
SDO2	Δ*vlpA*:: *pyr-4*; *alcA*(p)::gfp::kipA:: *pyr-4*	This study

*U. maydis*		
name	genotype	source
AB33	*a2 P_nar_:bW2bE1*	[76]
UMa299	*a2 P_nar_:bW2bE1 P_rrm4_:rrm4R_NatR P_otef_:tub1Gn_CbxR*	P. Becht
UMa781	*a2 P_nar_:bW2bE1 P_peb1_:peb1_NatR*	S. Baumann
UMa830	*a2 P_nar_:bW2bE1 P_rrm4_:rrm4C_HygR*	[35]
UMa958	*a2 P_nar_:bW2bE1 asp1*Δ*::HygR*	This study
UMa960	*a2 P_nar_:bW2bE1 P_asp1_:asp1G::HygR*	This study
UMa1112	*a2 P_nar_:bW2bE1 P_rrm4_:rrm4R_NatR P_otef_:tub1Gn_CbxR asp1Δ::HygR*	This study
UMa1158	*a2 P_nar_:bW2bE1 P_peb1_:peb1_NatR asp1Δ::HygR*	This study

### Generation of *asp1* variant containing plasmids and western blot analysis

*asp1^+^*, *asp1^1-364^* (plasmid p672), *asp1^H397A^* plasmids are derivatives of pJR2-3XL and have been described previously [7]. For the asp1^1-364^+asp1^365-920^ containing plasmid, p672 was cut with *SapI* and a PCR generated DNA fragment containing the *nmt1^+^* promoter followed by the DNA sequence encoding asp1^365-920^ inserted via homologous recombination in *S. cerevisiae* [83]. *asp1^R397A^* and *asp1^H807^* were generated by directed mutagenesis using the QuikChangeII Site-Directed Mutagenesis Kit (Stragene) and after verification of sequence by sequence analysis cloned into pJR-3XL via *S. cerevisiae* homologous recombination. To determine expression of plasmid-borne *asp1* variants, the appropriate *asp1* containing DNA sequences were fused to *gfp* and expression of the fusion protein was determined by western blot analysis as has been described [7]. *U. maydis* Vlp1G expression was determined via western blot analysis as has been described [36].

### Microscopy

For imaging of living *S. pombe* cells, cells were pre-grown in minimal medium at 25°C or 30°C and slides were prepared by mounting cells on agarose pads as described in [84]. Images were obtained at room temperature using a Zeiss Spinning Disc confocal microscope, equipped with a Yokogawa CSU-X1 unit and a MRm Camera. Slides were imaged using AxioVision software. Images shown are maximum intensity projections of 10–25 z-slices of 0.24–0.5 µm distance. For measurement of MT dynamics, strains expressing GFP-Atb2 [57] under control of the *nmt81* promoter were pre-grown under promoter-derepressing conditions for at least 48 hrs. For technical reasons, we used the *nmt81::gfp-atb2^+^* construct, as this facilitated the measurement of the sometimes faint MTs of the *asp1^D333A^* strain. Time-lapse images were acquired in 5–10 sec intervals.

For live-cell imaging of *A.nidulans* germlings and young hyphae, cells were grown on coverslips in 0.5 ml of Supplemented minimal media with 2% glycerol (de-repression of the *alcA* promoter, moderate induction). Cells were incubated at 30°C overnight/1 day. Coverslips were mounted on slide glass. Tempcontrol mini (Pepcon) was used for a constant temperature of the slide glass during microscopy. Images were captured using an Axiophot microscope using a Planapochromatic 63 times oil immersion objective lens, the Zeiss AxioCam MRM camera and the HBO103 mercury arc lamp (Osram) or HXP 120 (Zeiss, Jena, Germany). Images were collected and analyzed with the AxioVision system (Zeiss). Signal intensity was quantified with ImageJ software.

Live cell imaging of *U. maydis* was performed according to published protocols [36]. Microscope and camera were controlled by MetaMorph (Version 7.7.0.0, Molecular Devices, Seattle, IL, USA). The same software was used for measurements and image processing including the adjustment of brightness and contrast. MT bundles were visualized with a 63× Planapochromat (NA 1.4, Zeiss) or 100× α-Planapochromat (NA 1.46, Zeiss) in combination with a HXP lamp or laser illumination (488 nm), respectively. Z- stacks were composed of 38 planes with 270 nm spacing (63×) and 66 planes with 240 nm spacing (100×). Exposure time was 100 ms. Deconvolution was performed with Fiji. A theoretical PSF was determined with the diffraction PSF 3D plugin and images were generated using the Deconvolve 3D plugin [85], [86]. 3D movies were generated with MetaMorph. To determine the number of MT bundles z-stacks were collapsed to a maximum projection and after cytoplasmic background subtraction the number of bundles was determined. For determination of MT bundle intensity the maximum values of a longitudinal line scan (Fig. 7C) were plotted over distance. Each value from the x-axes was included in a whisker diagram (Fig. 7E) showing the median and range of fluorescent MT bundles (n = 10 cells for wild-type and *Umasp1*Δ, respectively). Fluorescence micrographs of *Umasp1-GFP* were acquired with 500 ms exposure time in a single plane. Before determining average cytoplasmic fluorescence images were background subtracted. For measurement of MT growth (Fig. 7F) strains expressing GFP-Tub1 were used. Z- stacks were composed three planes with 1 µm spacing (100× objective). Exposure time was 100 ms. For measurement of MT residence time (Fig. 7G) strains expressing Peb1-GFP were used. Z- stacks were composed of 5 planes with 800 nm spacing (100× objective). Exposure time was 100 ms. Statistical analysis was done with Prism5 (Graphpad).

## Supporting Information

Figure S1 Asp1 converts IP_6_ to IP_7_ *in vitro*. (A) Left panel: *S. cerevisiae* Vip1 for which enzymatic activity had been demonstrated was used as a positive control for IP_7_ generation [43]. 1 µg bacterially expressed and purified GST-Vip1^1-535^ (contains kinase domain) was used in an enzymatic reaction as described [54] followed by resolution of the products via PAGE and staining of the gel with Toluidine Blue. −, component not present in assay; +, component present in assay. Right panel: Asp1 generates IP_7_ from IP_6_ in an ATP-dependent reaction. 1 µg bacterially expressed and purified GST-Asp1 was used in the above mentioned *in vitro* assay. (B) Correlation between Asp1 protein input and the amount of IP_7_ generated. Left panel: Toluidine Blue stained PAGE showing IP_7_ produced by varying amounts of GST-Asp1 protein. Incubation time: 16 hrs. Right panel: Diagrammatic representation of the quantification of the IP_7_ bands shown in the left panel.
(TIF)

Figure S2 (A) *asp1^D333A^*, *asp1*Δ and *asp1^D333A, H397A^* strains show TBZ hypersensitivity. Serial dilution patch tests (10^5^–10^1^ cells) of the indicated strains on YE5S plates with (+) or (−) without TBZ. Plates were incubated for 5 days at 25°C. (B) *asp1^D333A^* and *asp1*Δ strains are sensitive to NaCl and caspofungin and resistant to treatment by the cell wall enzyme zymolyase. Serial dilution patch tests (10^5^–10^1^ cells) on YE5S plates with (+) or without (−) 50 mM NaCl or 1.5 µg/ml caspofungin, respectively. Plates were incubated for 4 days at 25°C. For zymolyase experiments cells were incubated with zymolyase and OD_600_ determined at the indicated time intervals. Reduction in OD_600_ is due to cell lysis.
(TIF)

Figure S3 Expression of plasmid-borne *asp1* variants in the *asp1*Δ strain. (A) Diagrammatic representation of the *S. pombe* LEU2 plasmids used in B–C. P, *nmt1^+^* promoter. (B) Western blot analysis of the *asp1*Δ strain expressing the indicated Asp1-GFP variants. Similar amounts of protein were resolved by SDS-PAGE and probed with an anti-GFP antibody or an anti-γ-tubulin antibody (loading control). (C) Quantification and diagrammatic representation of the Asp1-GFP signals obtained in (B).
(TIF)

Figure S4 (A) Serial dilution patch tests (10^5^–10^1^ cells) of the indicated strains grown on minimal medium without thiamine (promoter on conditions) for 5 or 4 days at 25°C or 30°C, respectively. Incubation on TBZ containing plates was for 9 days at 25°C. (B) Live cell images of the indicated strains expressing *gfp-atb2^+^*. Time between the images is 10 seconds. In each case the arrow indicates a short MT that polymerizes from the cell middle but is not oriented along the long axis of the cell. In the wild-type strain this MT reaches the cell cortex (80 seconds image), becomes deflected and continues to grow. In the *asp1^D333A^* strain, such a MT touches the cell cortex (100 second image) and then depolymerizes. Bars, 5 µm.
(TIF)

Figure S5 (A) Sequence comparison of the Vip1 family members from *S. pombe*, *S. cerevisiae*, *A. nidulans* (AN5797.2) and *U. maydis* (UM06407.1). Multiple sequence alignment was performed with MultAlin using BLOSUM62 matrix [87]. (B) The respective kinase and phosphatase domains are indicated in green and grey, respectively.
(TIF)

Figure S6 GFP-KipA, a marker of growing MT plus-ends, in the wild-type (SSK92) and the *vlpA*-deletion strain (SDO2). (A) Diagrammatic representation of the components shown in (B) and (C). (B) and (C) We compared newly emanating GFP-KipA signals in the wild-type (B) and the *ΔvlpA* strain (C) during a 5 minute time period at SPBs (asterisks) and at septal-MTOC (white arrows). Bar, 10 µm. Kymographs at septa during a 5 minute time period are shown. GFP signals coming from the septum are shown by blue arrows. GFP signals arriving at the septum are shown by red arrows. Bar, 1 µm.
(TIF)

Figure S7 Loss of UmAsp1 causes defects in proliferation and leads to TBZ sensitivity. (A) Growth of the indicated yeast strains over time. (B) Serial dilution patch test (10^7^ to 10^5^ cells) of the indicated strains grown with/without 10 µg/ml TBZ. (C) Filter paper with/without 10 µg/ml TBZ was placed on a lawn of *U. maydis* cells (strains indicated above). The region indicated by a white bar was measured to determine the zone of inhibition (radius in cm) given in Figure 7A. Note, plates of *Umasp1*Δ cells appear slightly darker due to secretion of an unknown pigment.
(TIF)

Figure S8 Loss of UmAsp1 causes alterations in cell morphology. Representative DIC images of wild-type (A) and *Umasp1*Δ (B) cells, quantified in Figure 7B are shown (Bars, 10 µm).
(TIF)

Figure S9 Loss of UmAsp1 causes defects in MT organization. Representative fluorescence images of wild-type (A) and *Umasp1*Δ (B) cells are shown. (C) The indicated MT categories were determined in wild-type and *Umasp1*Δ strains. Bars show the mean of three independent experiments with n>100 cells (error bars show SEM, p<0.0001; two-way ANOVA test).
(TIF)

Figure S10 Loss of UmAsp1 causes defects in filamentous growth. Representative DIC images of wild-type (A) and *Umasp1*Δ (B) hyphae 8 hours after filament inducing conditions (Bars, 10 µm). Quantification is shown in Figure 8C.
(TIF)

Figure S11 UmAsp1-GFP signal decreases during switch to filamentous growth. Fluorescence micrographs of mixed cultures expressing either UmAsp1-GFP (*) or Rrm4-mCherry (#) [35] are shown. Micrographs detecting either green or red fluorescence were taken subsequently from the same region of interest: (A) yeast, (B) filaments. Thereby, the degree of green auto-fluorescence (seen in the Rrm4-mCherry control) can be judged.
(TIF)

Figure S12 (A) Time dependent generation of IP_7_ by GST-Asp1 variants. 4 µg of the indicated proteins were used in an ATP-dependent enzymatic reaction and the resulting inositol pyrophosphates were resolved on a 35,5% PAGE and stained with Toluidine Blue. −, component not present; +, component present. (B) Quantification and diagrammatic representation of the IP_7_ bands obtained in the assay shown in (A).
(TIF)

Figure S13 IP_6_ amounts in the presence (+) or absence (−) of 9 µg Asp1^365-920^. Assay conditions and detection of IP_6_ were as described for the *in vitro* kinase assay.
(TIF)

Figure S14 Western blot analysis of the *asp1*Δ strain expressing the indicated Asp1-GFP (arrow shows full length fusion protein) variants. Similar amounts of protein were resolved by SDS-PAGE and probed with an anti-GFP antibody or an anti-γ-tubulin antibody (left and right panels, respectively).
(TIF)

Figure S15 Percentage of MTs polymerizing towards the lateral cortex (black bars) or towards a cell end (white bars). Wild-type: n = 77, *asp1^H397A^*: n = 73, *asp1^D333A^* n = 83.
(TIF)

Figure S16 Diagrammatic representation of the number of interphase MTs in the indicated strains (*mal3*Δ strain, n = 95; *mal3*Δ *asp1^H397A^* strain, n = 99).
(TIF)

Figure S17 Movement of outmost outbound Tea2-GFP comets (see diagram). Speed of comets (nm/sec): wild-type, 60±30, n = 89; *asp1^H397A^*, 60±26,7, n = 64; *asp1^D333A^*, 90±43,3, n = 71. * p<0.0005 for *asp1^D333A^* vs. wild-type (Welch-test).
(TIF)

Figure S18 Generation of IP_7_ by GST-Asp1 with varying amounts (2,4,8 µg) of Asp1^365-920 H397A^. Enzymatic reaction was carried out as described in Figure 1C. −, component not present; +, component present.
(TIF)

Movie S1 GFP-KipA, a marker of growing MT plus-ends in the wild-type strain (SSK92). 2 seconds intervals, total 5 minutes. Scale bar, 10 µm.
(AVI)

Movie S2 GFP-KipA in the *vlpA*-deletion strain (SDO2). 2 seconds intervals, total 5 minutes. Scale bar, 10 µm.
(AVI)

Movie S3 3D reconstruction of a wild-type cell expressing GFP-Tub1. The underlying z-stack is depicted in Figure 7. Size of angle images of the z-stack was doubled and pixels resampled. Ratio of xy-distance and xz-distance was chosen 1∶1 to obtain cubic voxels. Movie comprises 14 frames in 12 seconds.
(MOV)

Movie S4 3D reconstruction as in Movie S3 of an *Umasp1*Δ cell expressing GFP-Tub1.
(MOV)
