# Supplementary figures and images for: The Vip1 Inositol Polyphosphate Kinase Family Regulates Polarized Growth and Modulates the Microtubule Cytoskeleton in Fungi
Source: PLoS Genet. 2014 Sep 25;10(9):e1004586. doi: 10.1371/journal.pgen.1004586 (PMC4177672; doi:10.1371/journal.pgen.1004586)

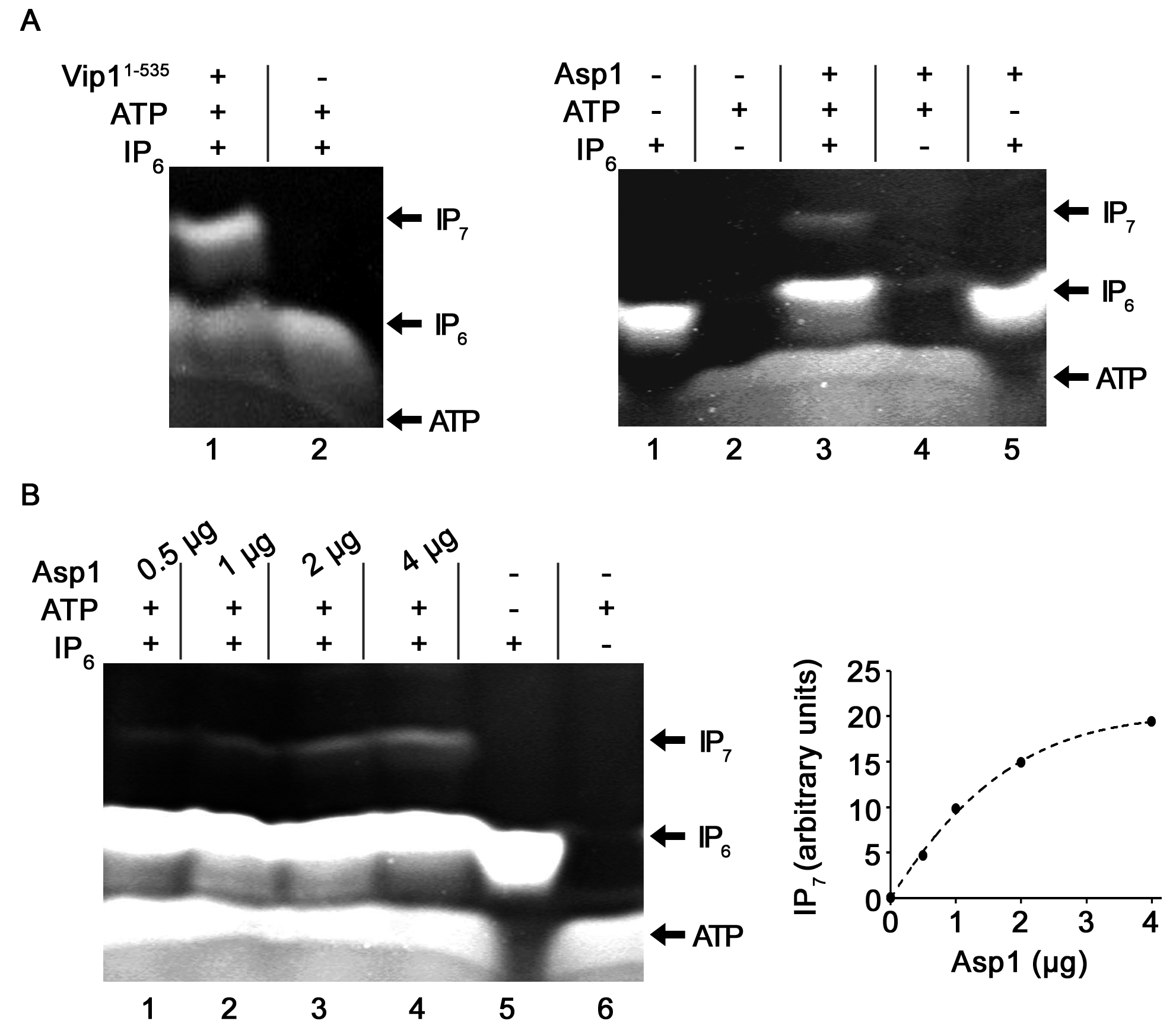

Supplement: Figure S1 — Asp1 converts IP6 to IP7 in vitro. (A) Left panel: S. cerevisiae Vip1 for which enzymatic activity had been demonstrated was used as a positive control for IP7 generation [43]. 1 µg bacterially expressed and purified GST-Vip11-535 (contains kinase domain) was used in an enzymatic reaction as described [54] followed by resolution of the products via PAGE and staining of the gel with Toluidine Blue. −, component not present in assay; +, component present in assay. Right panel: Asp1 generates IP7 from IP6 in an ATP-dependent reaction. 1 µg bacterially expressed and purified GST-Asp1 was used in the above mentioned in vitro assay. (B) Correlation between Asp1 protein input and the amount of IP7 generated. Left panel: Toluidine Blue stained PAGE showing IP7 produced by varying amounts of GST-Asp1 protein. Incubation time: 16 hrs. Right panel: Diagrammatic representation of the quantification of the IP7 bands shown in the left panel. (TIF) [file pgen.1004586.s001.tif]

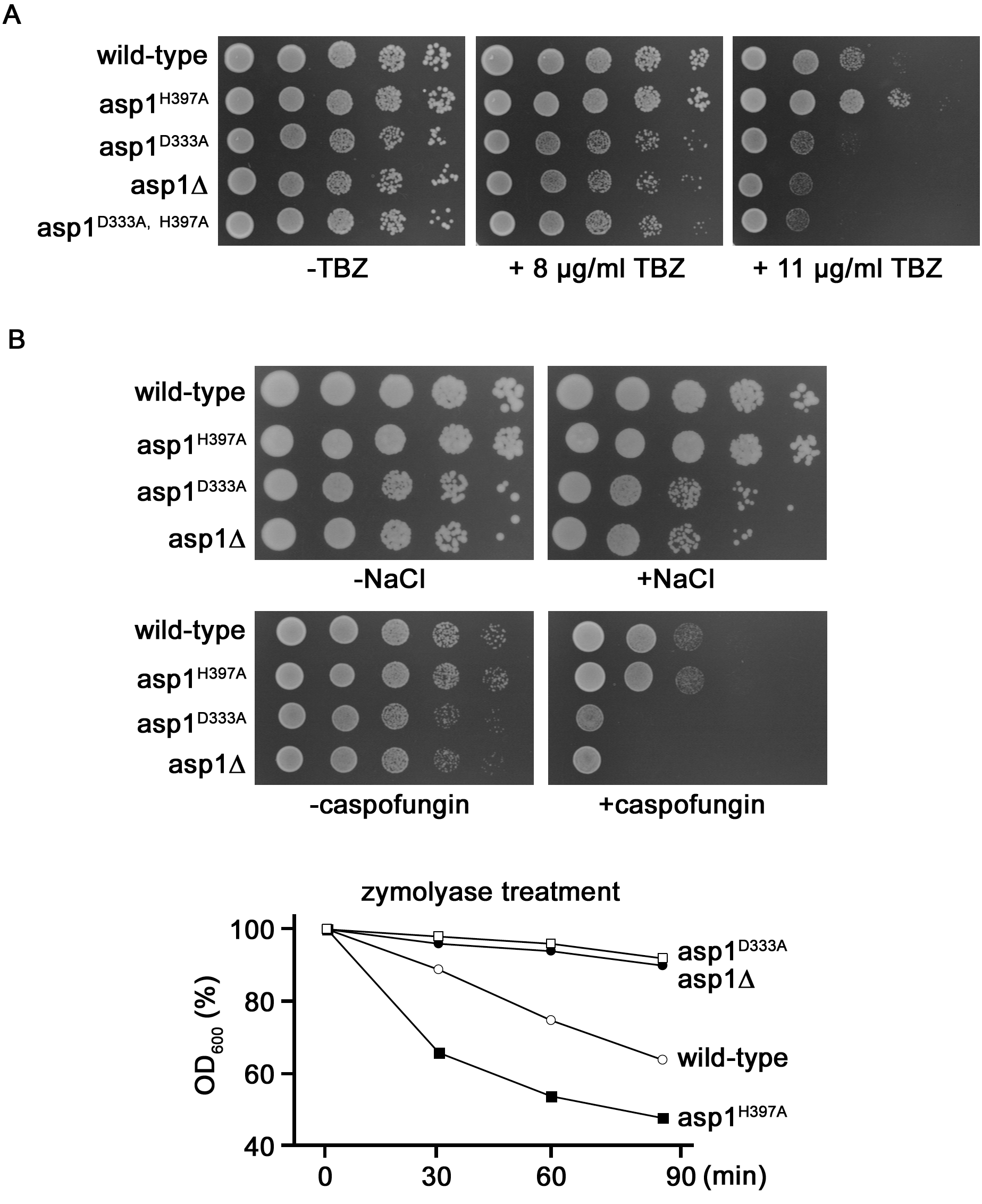

Supplement: Figure S2 — (A) asp1D333A, asp1Δ and asp1D333A, H397A strains show TBZ hypersensitivity. Serial dilution patch tests (105–101 cells) of the indicated strains on YE5S plates with (+) or (−) without TBZ. Plates were incubated for 5 days at 25°C. (B) asp1D333A and asp1Δ strains are sensitive to NaCl and caspofungin and resistant to treatment by the cell wall enzyme zymolyase. Serial dilution patch tests (105–101 cells) on YE5S plates with (+) or without (−) 50 mM NaCl or 1.5 µg/ml caspofungin, respectively. Plates were incubated for 4 days at 25°C. For zymolyase experiments cells were incubated with zymolyase and OD600 determined at the indicated time intervals. Reduction in OD600 is due to cell lysis. (TIF) [file pgen.1004586.s002.tif]

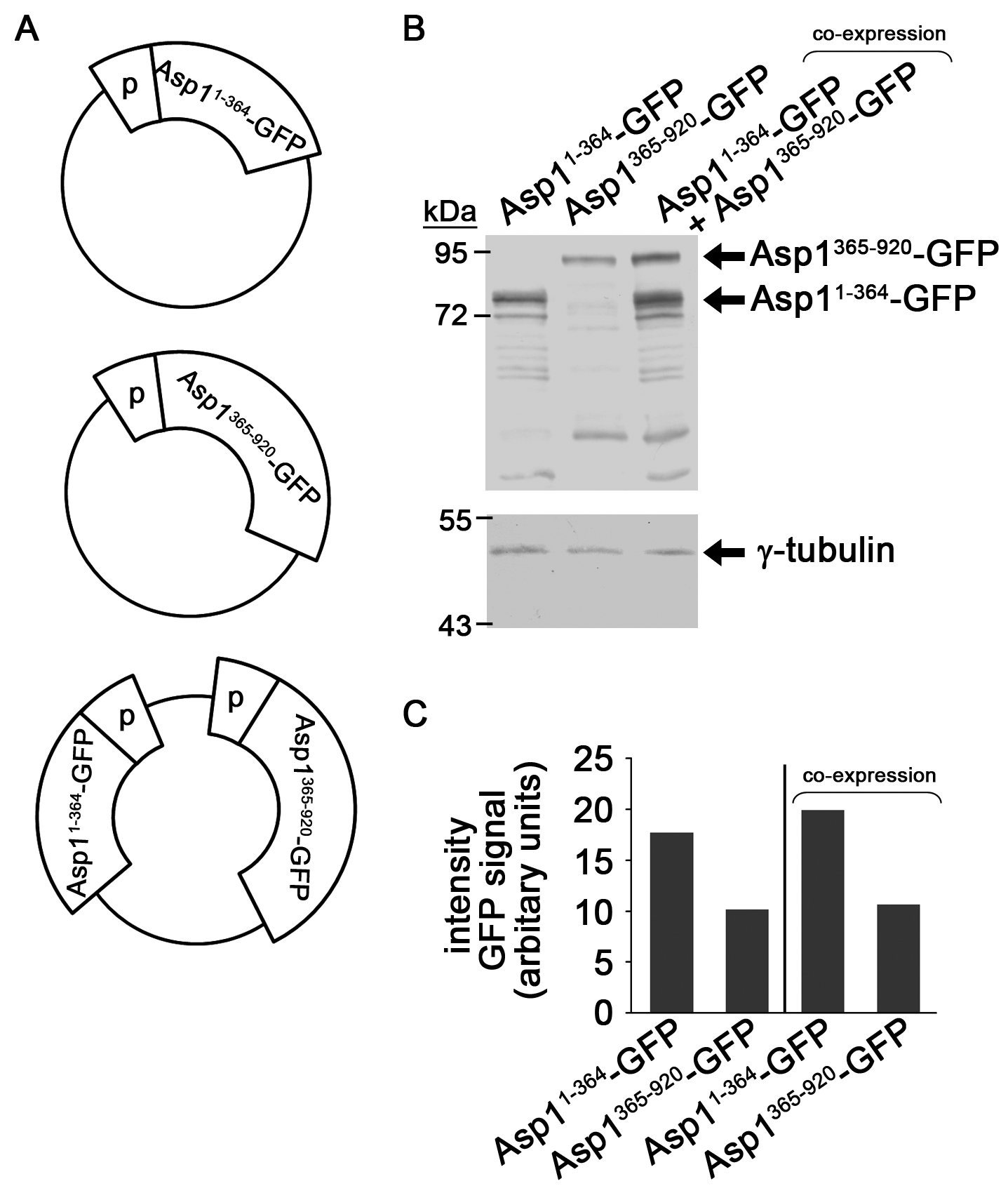

Supplement: Figure S3 — Expression of plasmid-borne asp1 variants in the asp1Δ strain. (A) Diagrammatic representation of the S. pombe LEU2 plasmids used in B–C. P, nmt1+ promoter. (B) Western blot analysis of the asp1Δ strain expressing the indicated Asp1-GFP variants. Similar amounts of protein were resolved by SDS-PAGE and probed with an anti-GFP antibody or an anti-γ-tubulin antibody (loading control). (C) Quantification and diagrammatic representation of the Asp1-GFP signals obtained in (B). (TIF) [file pgen.1004586.s003.tif]

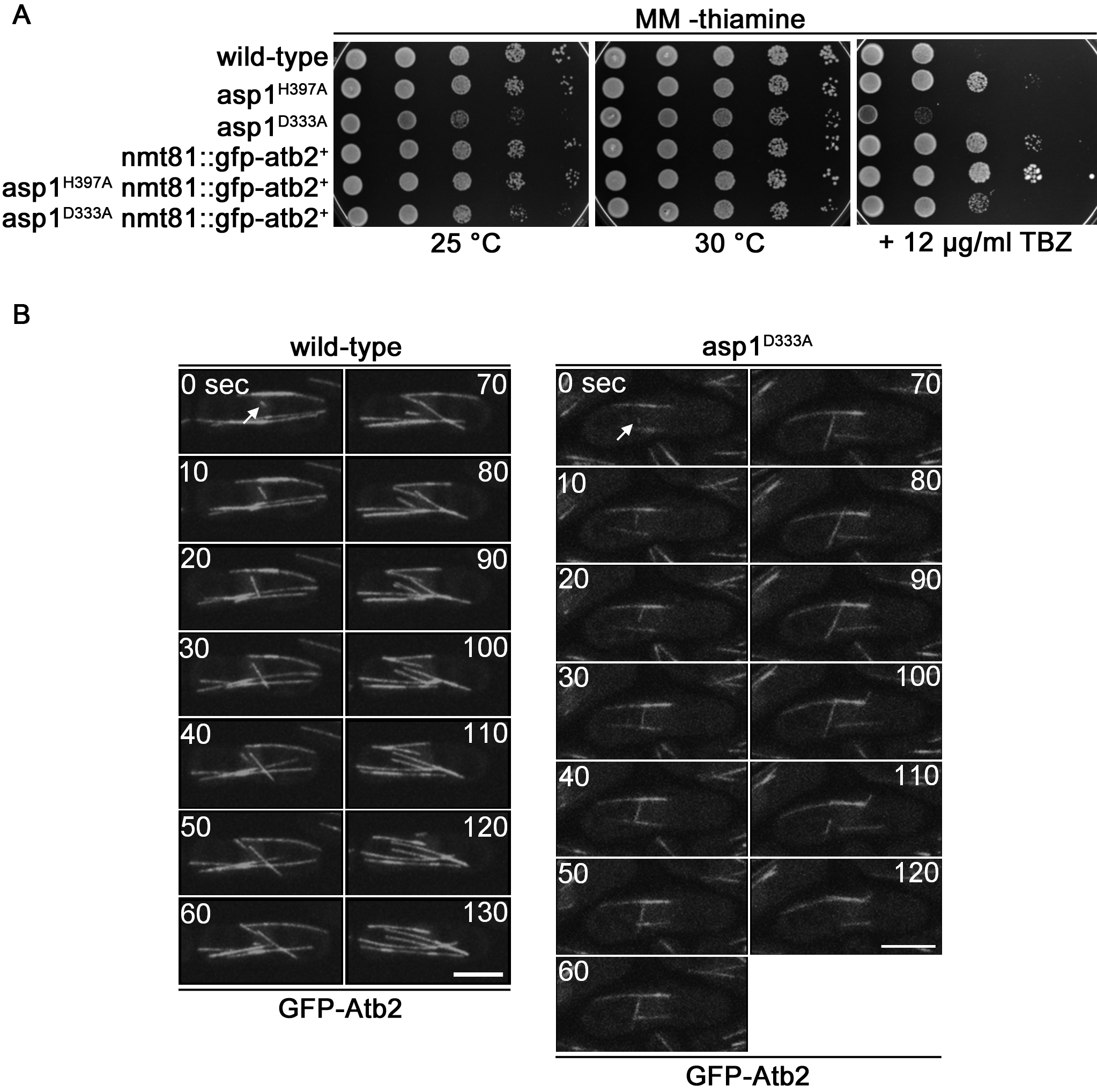

Supplement: Figure S4 — (A) Serial dilution patch tests (105–101 cells) of the indicated strains grown on minimal medium without thiamine (promoter on conditions) for 5 or 4 days at 25°C or 30°C, respectively. Incubation on TBZ containing plates was for 9 days at 25°C. (B) Live cell images of the indicated strains expressing gfp-atb2+. Time between the images is 10 seconds. In each case the arrow indicates a short MT that polymerizes from the cell middle but is not oriented along the long axis of the cell. In the wild-type strain this MT reaches the cell cortex (80 seconds image), becomes deflected and continues to grow. In the asp1D333A strain, such a MT touches the cell cortex (100 second image) and then depolymerizes. Bars, 5 µm. (TIF) [file pgen.1004586.s004.tif]

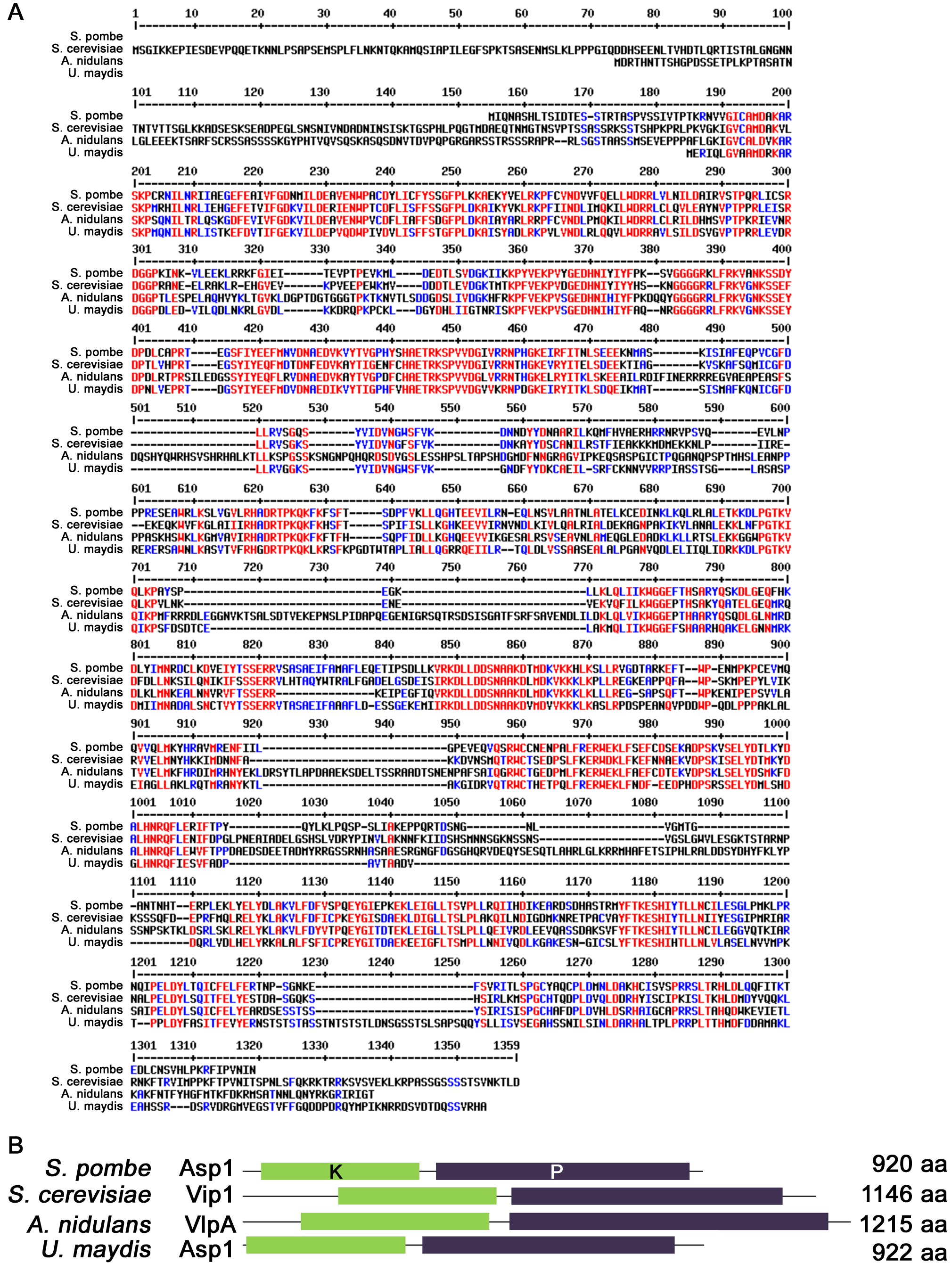

Supplement: Figure S5 — (A) Sequence comparison of the Vip1 family members from S. pombe, S. cerevisiae, A. nidulans (AN5797.2) and U. maydis (UM06407.1). Multiple sequence alignment was performed with MultAlin using BLOSUM62 matrix [87]. (B) The respective kinase and phosphatase domains are indicated in green and grey, respectively. (TIF) [file pgen.1004586.s005.tif]

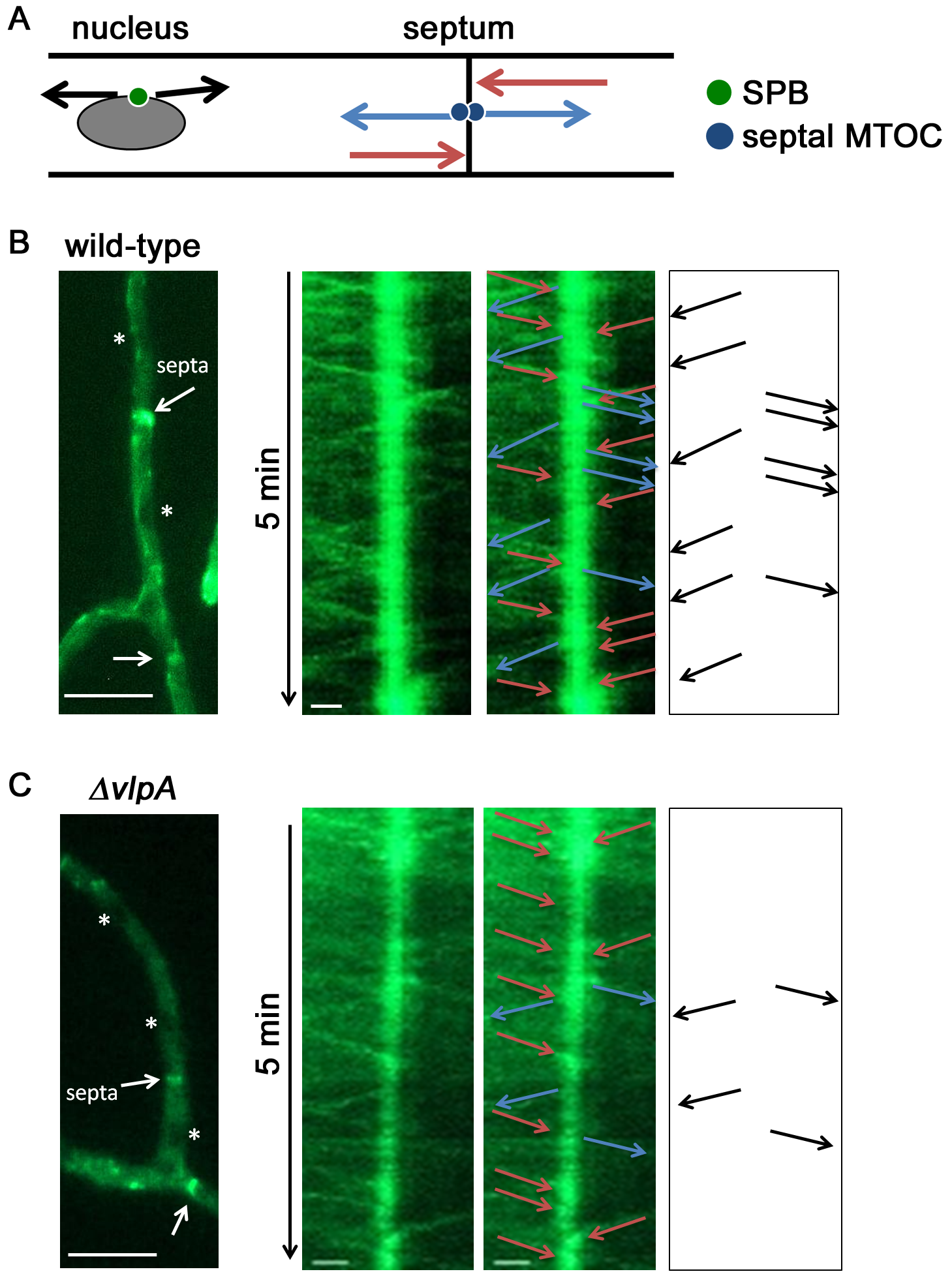

Supplement: Figure S6 — GFP-KipA, a marker of growing MT plus-ends, in the wild-type (SSK92) and the vlpA-deletion strain (SDO2). (A) Diagrammatic representation of the components shown in (B) and (C). (B) and (C) We compared newly emanating GFP-KipA signals in the wild-type (B) and the ΔvlpA strain (C) during a 5 minute time period at SPBs (asterisks) and at septal-MTOC (white arrows). Bar, 10 µm. Kymographs at septa during a 5 minute time period are shown. GFP signals coming from the septum are shown by blue arrows. GFP signals arriving at the septum are shown by red arrows. Bar, 1 µm. (TIF) [file pgen.1004586.s006.tif]

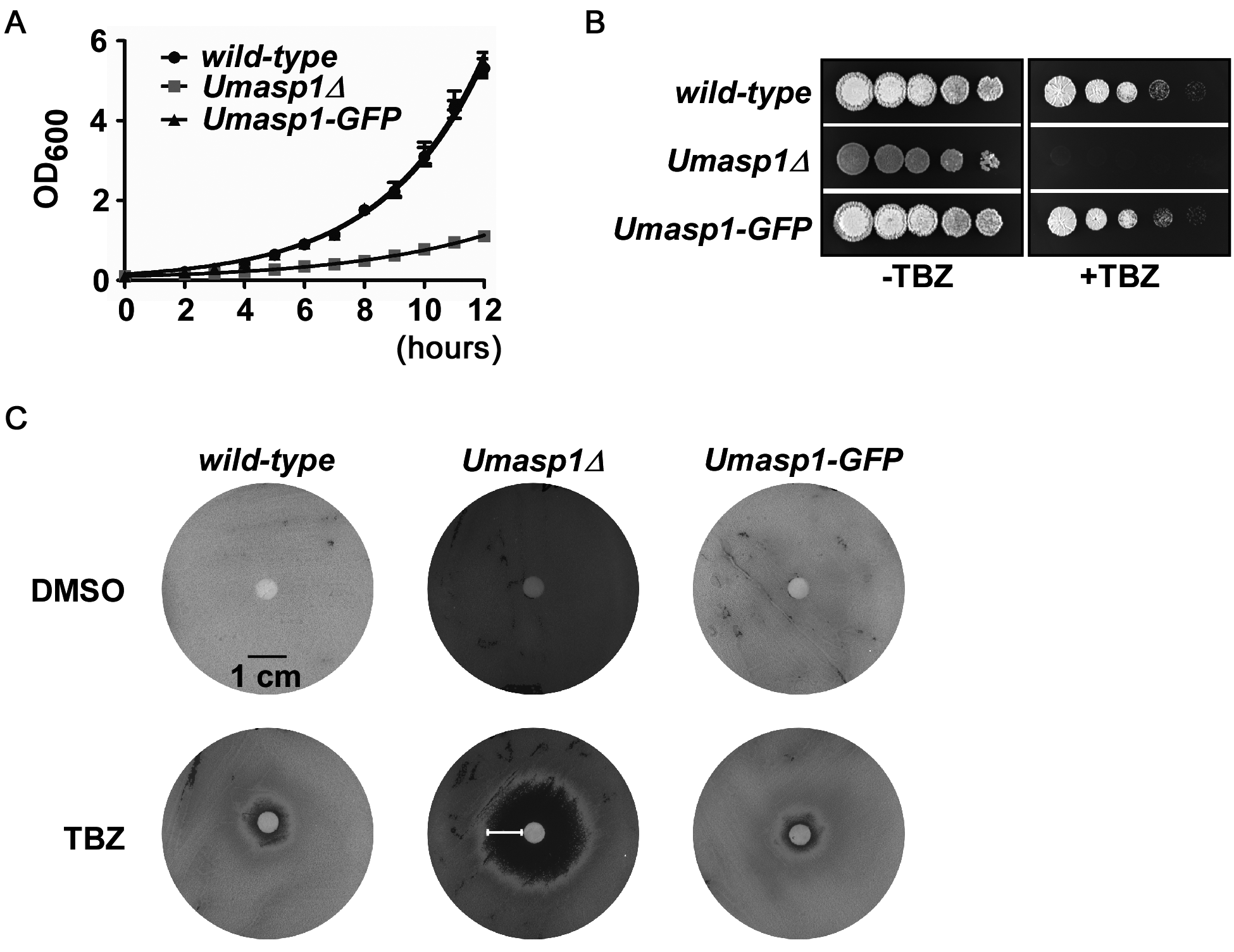

Supplement: Figure S7 — Loss of UmAsp1 causes defects in proliferation and leads to TBZ sensitivity. (A) Growth of the indicated yeast strains over time. (B) Serial dilution patch test (107 to 105 cells) of the indicated strains grown with/without 10 µg/ml TBZ. (C) Filter paper with/without 10 µg/ml TBZ was placed on a lawn of U. maydis cells (strains indicated above). The region indicated by a white bar was measured to determine the zone of inhibition (radius in cm) given in Figure 7A. Note, plates of Umasp1Δ cells appear slightly darker due to secretion of an unknown pigment. (TIF) [file pgen.1004586.s007.tif]

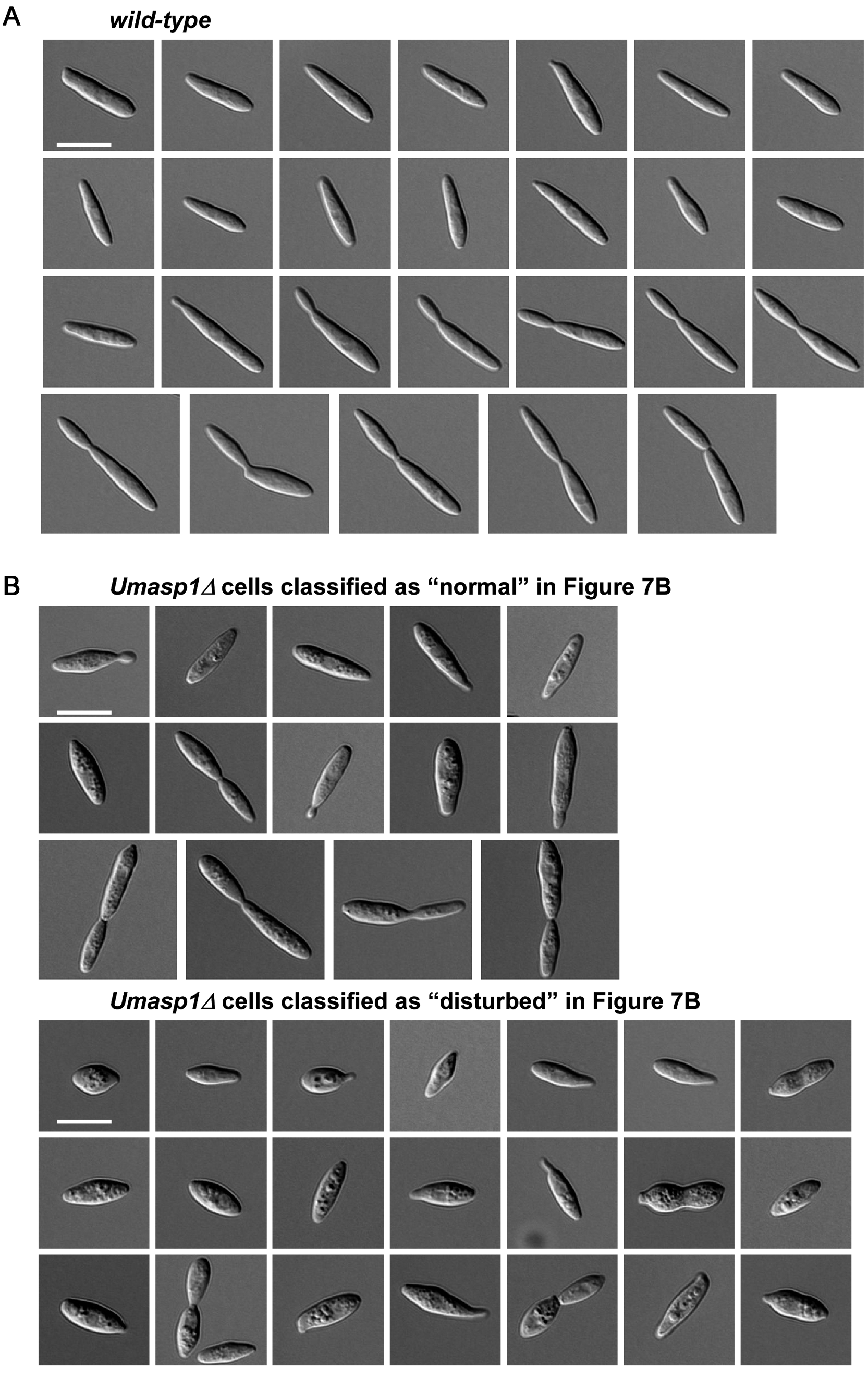

Supplement: Figure S8 — Loss of UmAsp1 causes alterations in cell morphology. Representative DIC images of wild-type (A) and Umasp1Δ (B) cells, quantified in Figure 7B are shown (Bars, 10 µm). (TIF) [file pgen.1004586.s008.tif]

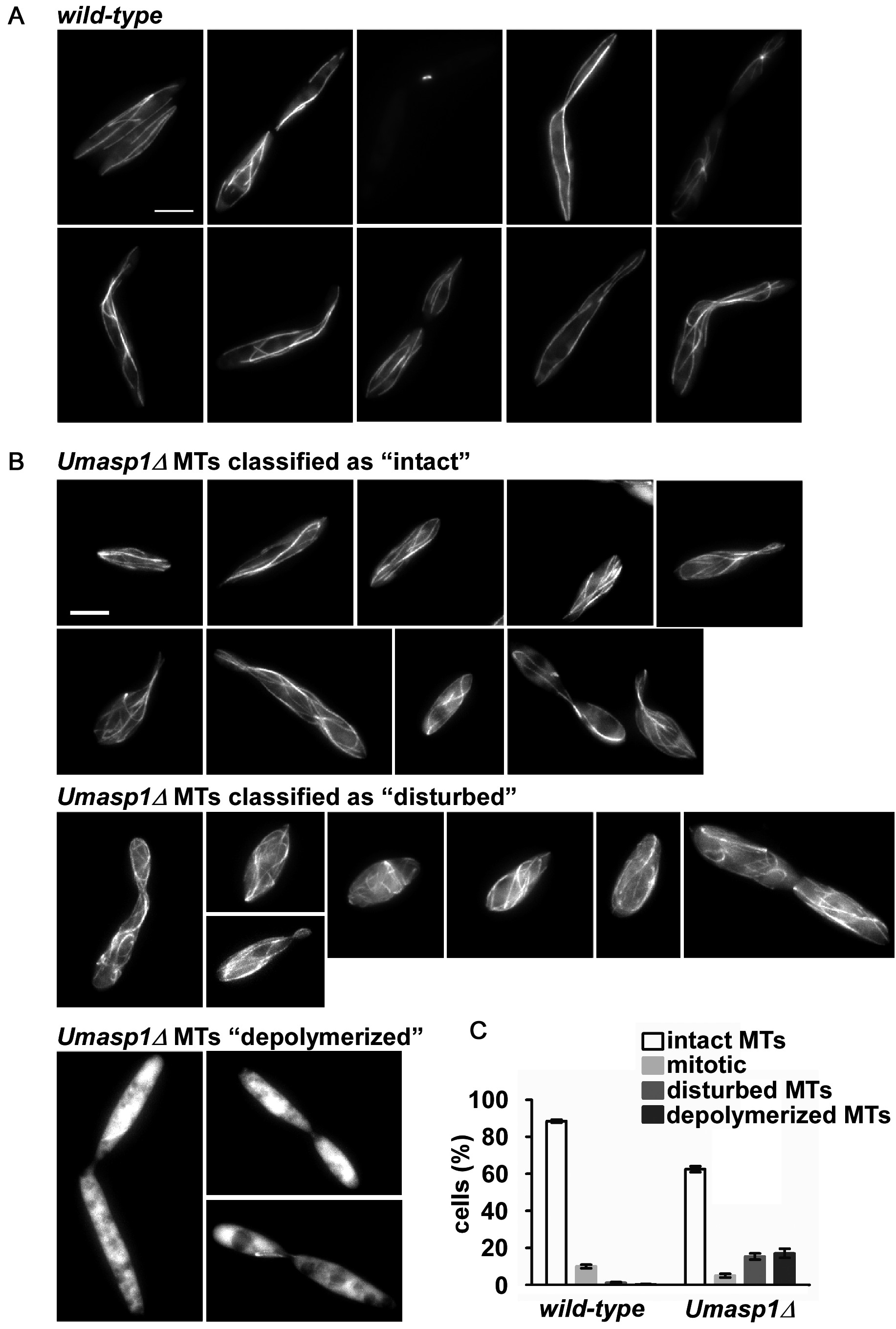

Supplement: Figure S9 — Loss of UmAsp1 causes defects in MT organization. Representative fluorescence images of wild-type (A) and Umasp1Δ (B) cells are shown. (C) The indicated MT categories were determined in wild-type and Umasp1Δ strains. Bars show the mean of three independent experiments with n>100 cells (error bars show SEM, p<0.0001; two-way ANOVA test). (TIF) [file pgen.1004586.s009.tif]

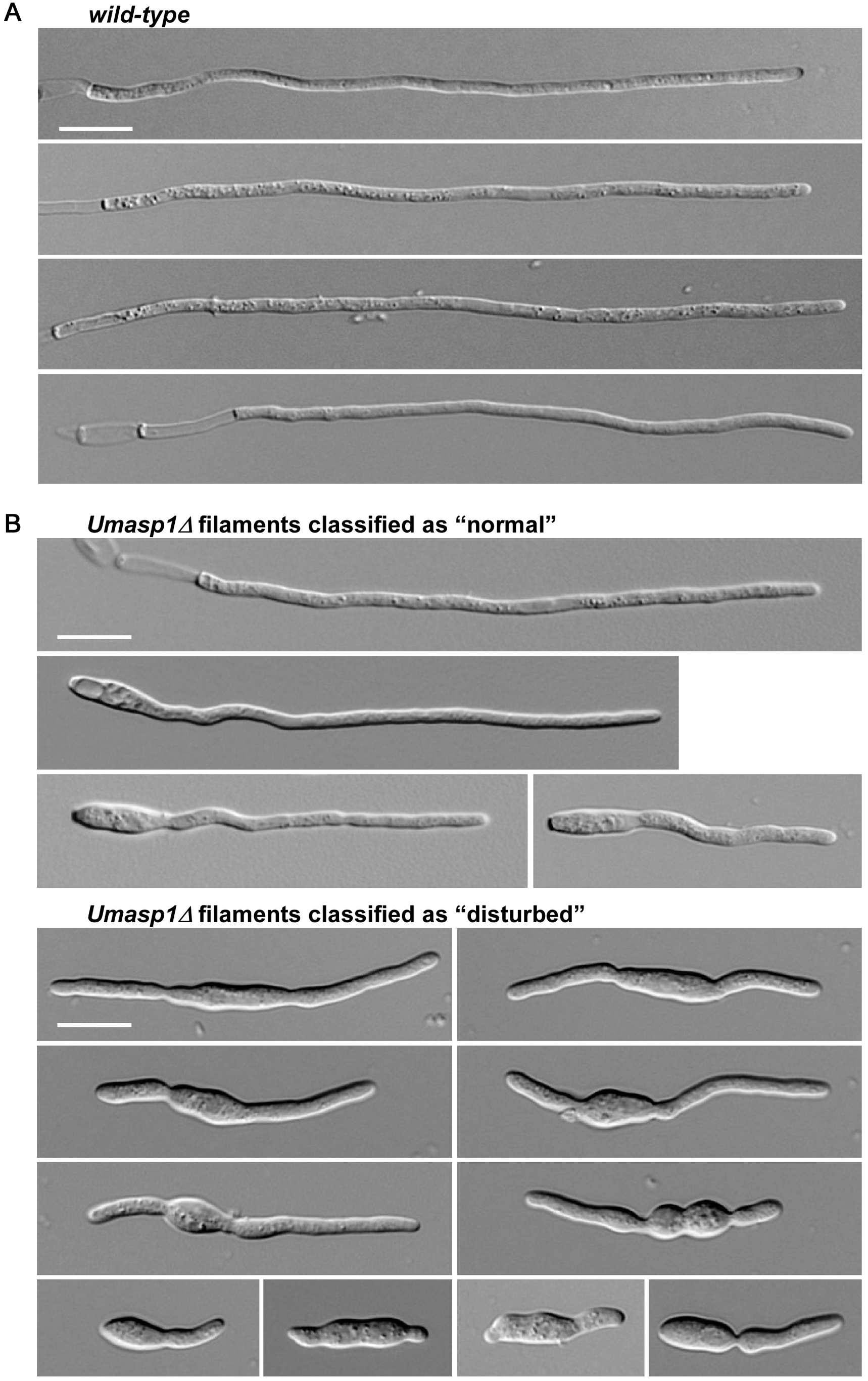

Supplement: Figure S10 — Loss of UmAsp1 causes defects in filamentous growth. Representative DIC images of wild-type (A) and Umasp1Δ (B) hyphae 8 hours after filament inducing conditions (Bars, 10 µm). Quantification is shown in Figure 8C. (TIF) [file pgen.1004586.s010.tif]

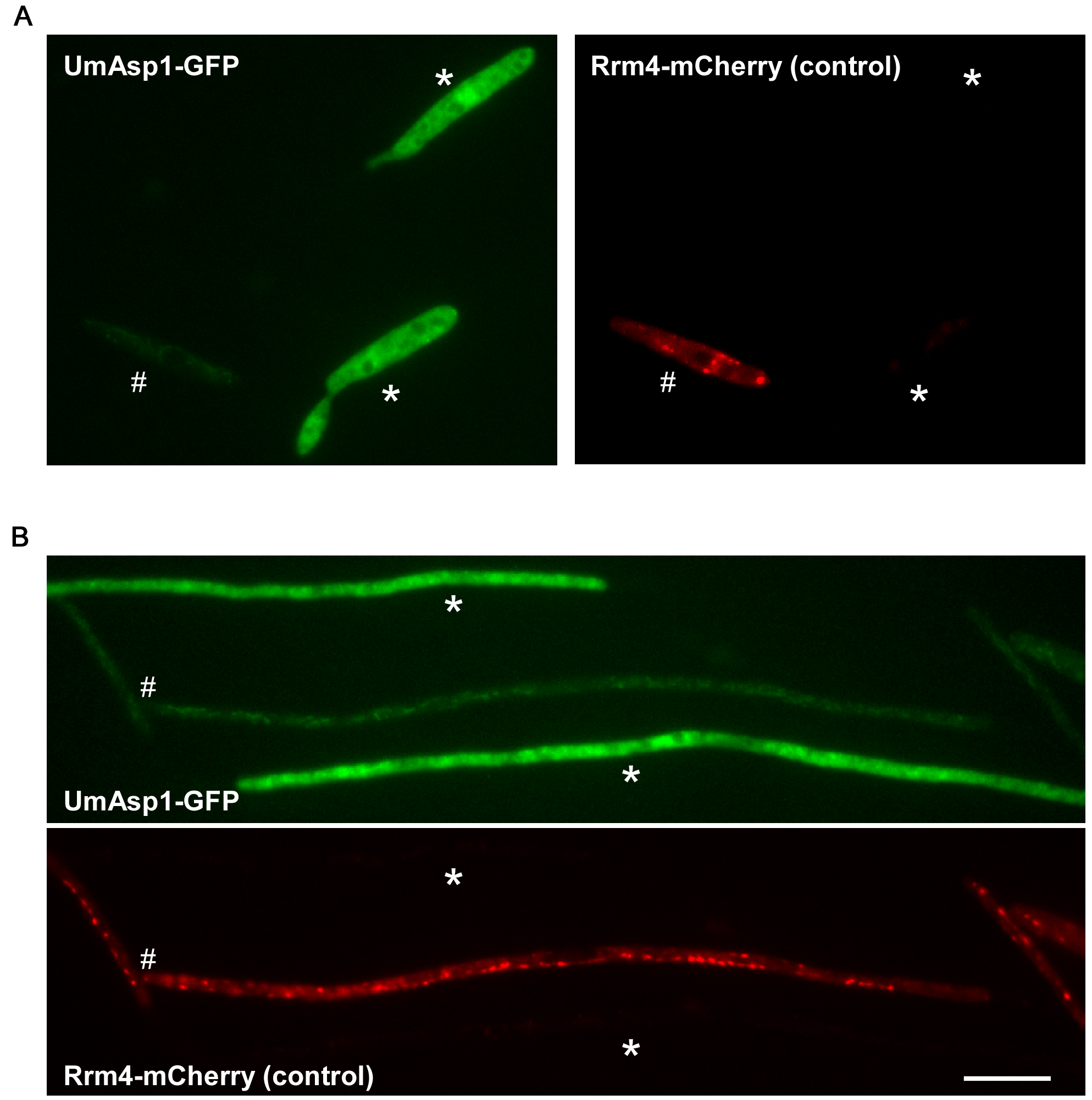

Supplement: Figure S11 — UmAsp1-GFP signal decreases during switch to filamentous growth. Fluorescence micrographs of mixed cultures expressing either UmAsp1-GFP (*) or Rrm4-mCherry (#) [35] are shown. Micrographs detecting either green or red fluorescence were taken subsequently from the same region of interest: (A) yeast, (B) filaments. Thereby, the degree of green auto-fluorescence (seen in the Rrm4-mCherry control) can be judged. (TIF) [file pgen.1004586.s011.tif]

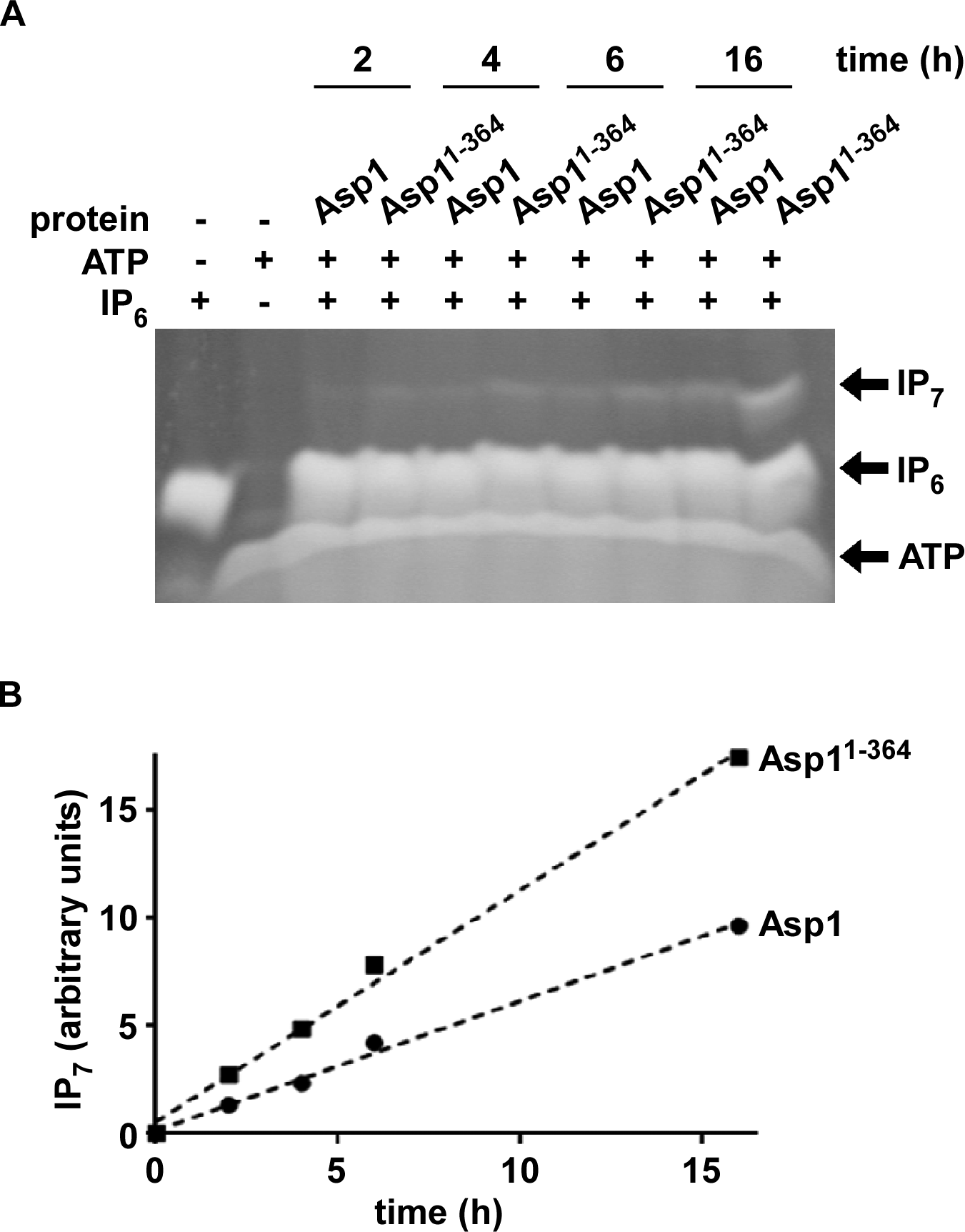

Supplement: Figure S12 — (A) Time dependent generation of IP7 by GST-Asp1 variants. 4 µg of the indicated proteins were used in an ATP-dependent enzymatic reaction and the resulting inositol pyrophosphates were resolved on a 35,5% PAGE and stained with Toluidine Blue. −, component not present; +, component present. (B) Quantification and diagrammatic representation of the IP7 bands obtained in the assay shown in (A). (TIF) [file pgen.1004586.s012.tif]

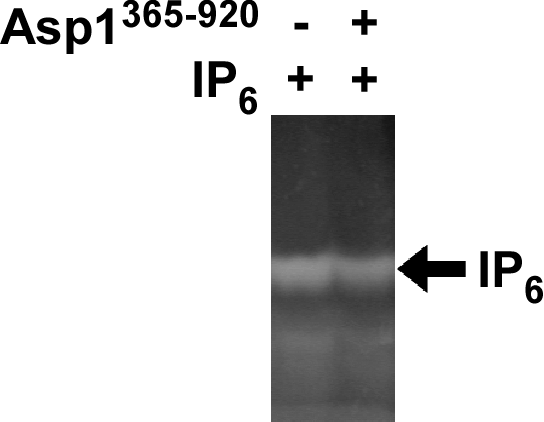

Supplement: Figure S13 — IP6 amounts in the presence (+) or absence (−) of 9 µg Asp1365-920. Assay conditions and detection of IP6 were as described for the in vitro kinase assay. (TIF) [file pgen.1004586.s013.tif]

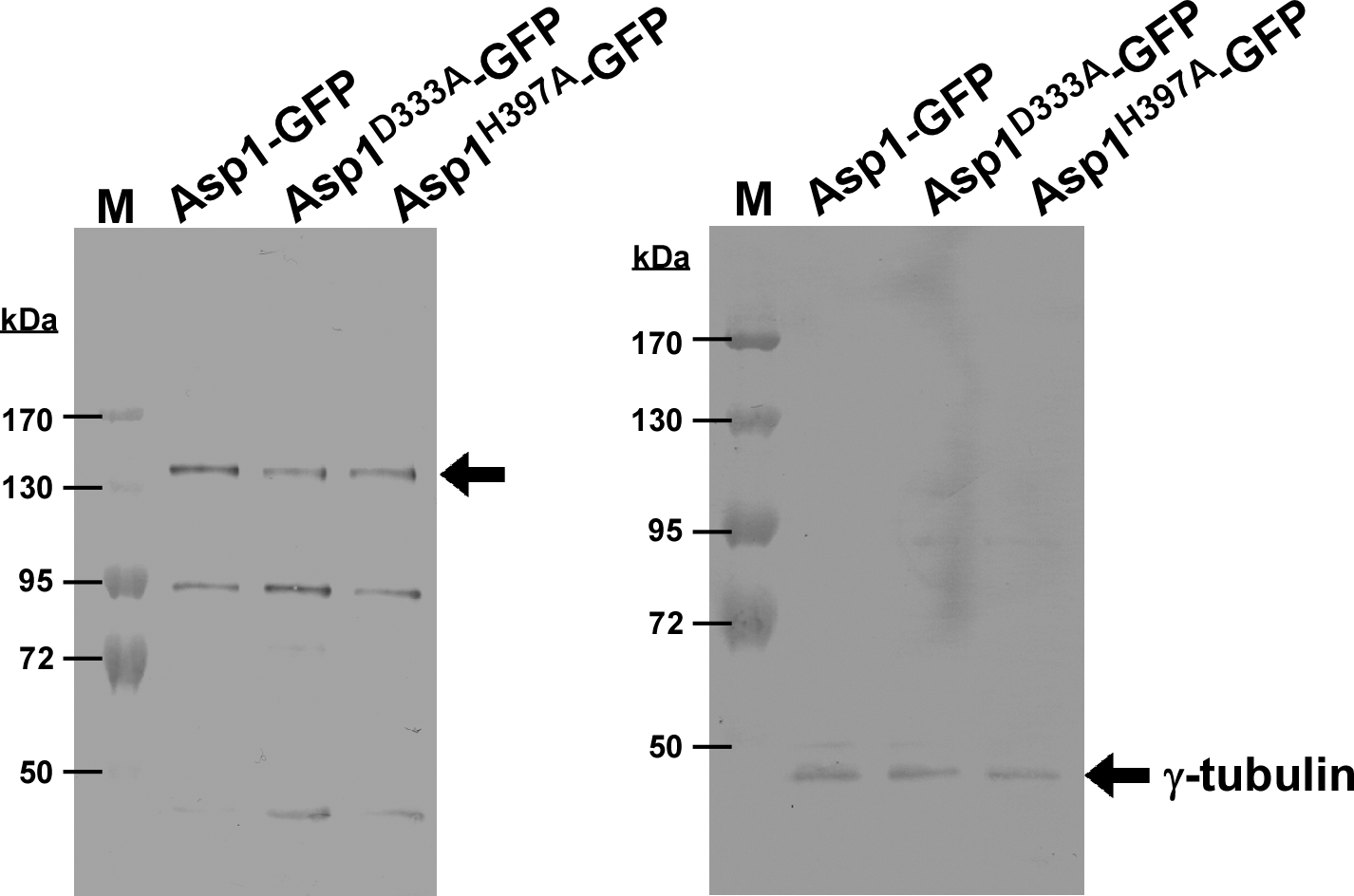

Supplement: Figure S14 — Western blot analysis of the asp1Δ strain expressing the indicated Asp1-GFP (arrow shows full length fusion protein) variants. Similar amounts of protein were resolved by SDS-PAGE and probed with an anti-GFP antibody or an anti-γ-tubulin antibody (left and right panels, respectively). (TIF) [file pgen.1004586.s014.tif]

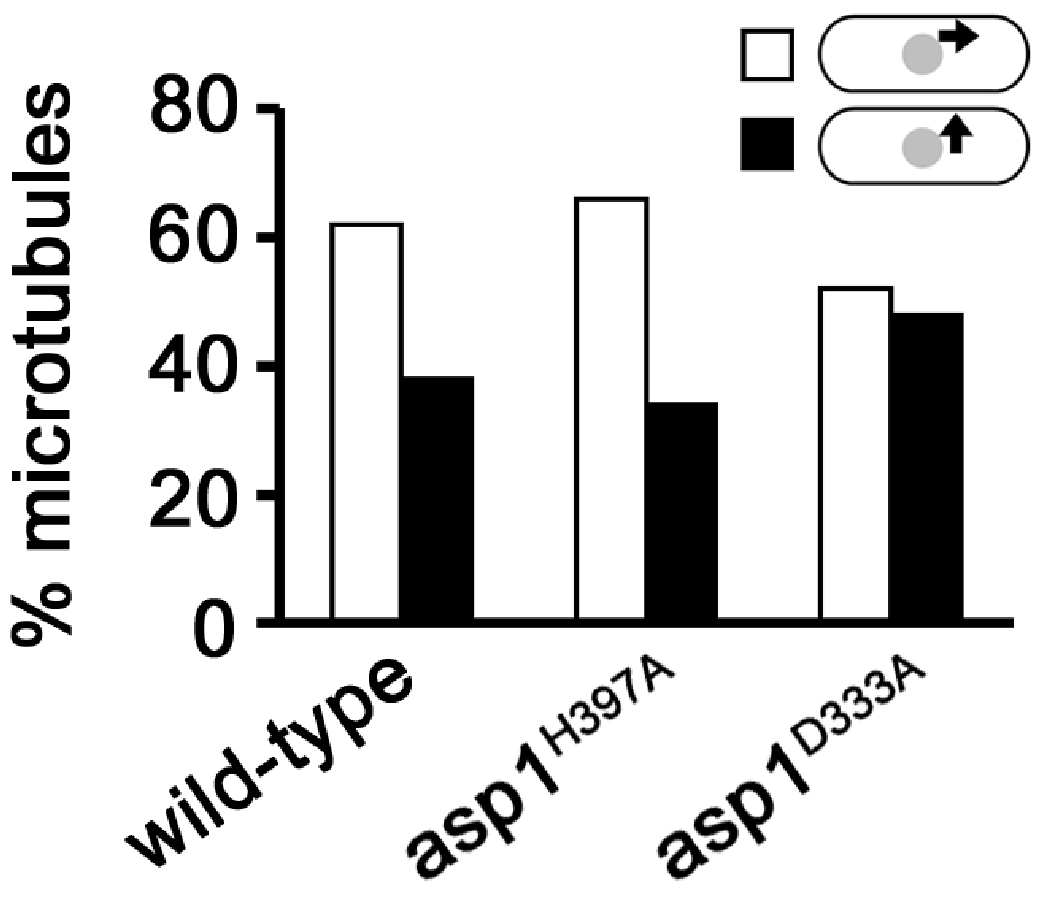

Supplement: Figure S15 — Percentage of MTs polymerizing towards the lateral cortex (black bars) or towards a cell end (white bars). Wild-type: n = 77, asp1H397A: n = 73, asp1D333A n = 83. (TIF) [file pgen.1004586.s015.tif]

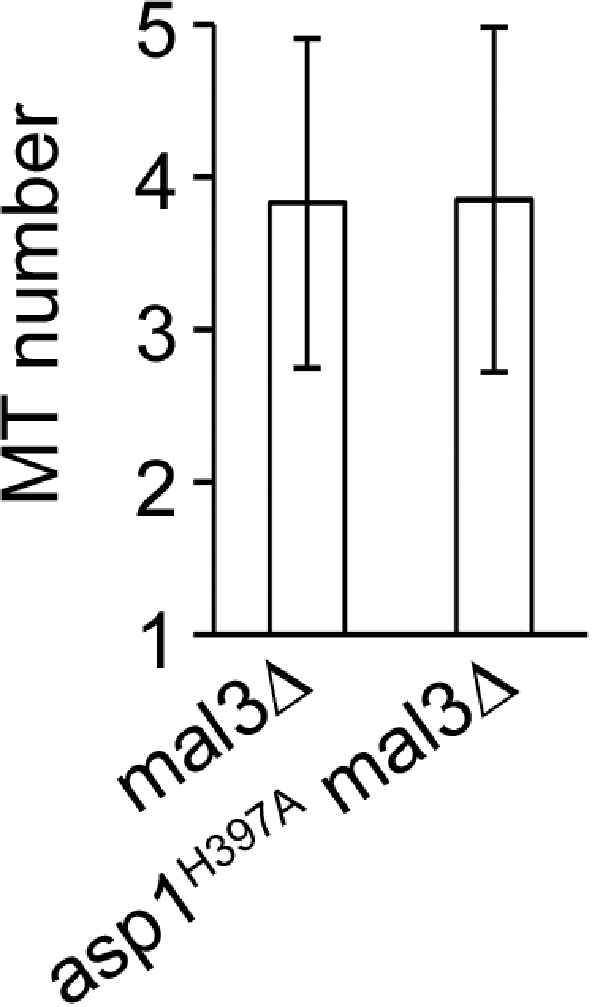

Supplement: Figure S16 — Diagrammatic representation of the number of interphase MTs in the indicated strains (mal3Δ strain, n = 95; mal3Δ asp1H397A strain, n = 99). (TIF) [file pgen.1004586.s016.tif]

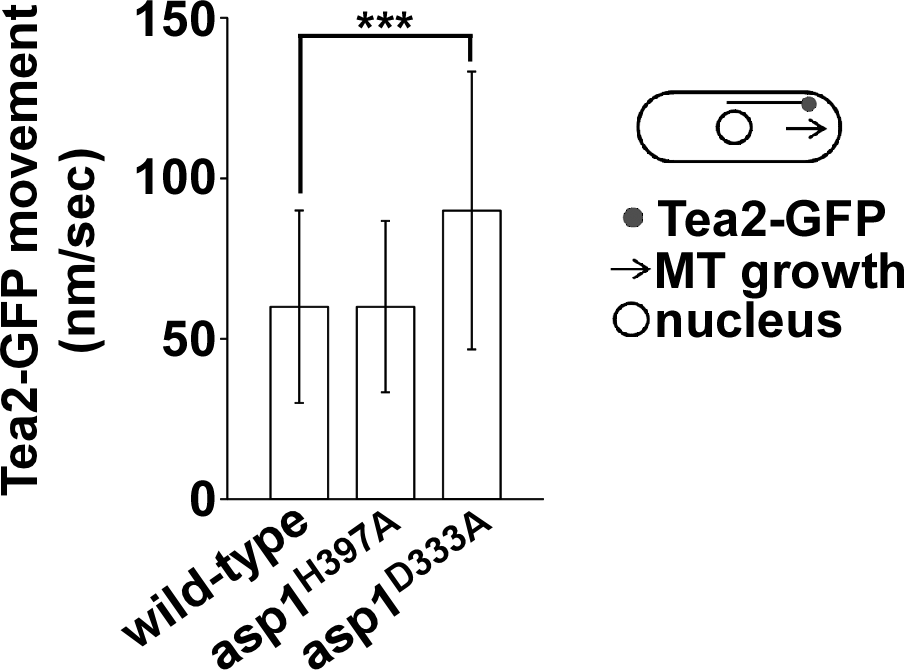

Supplement: Figure S17 — Movement of outmost outbound Tea2-GFP comets (see diagram). Speed of comets (nm/sec): wild-type, 60±30, n = 89; asp1H397A, 60±26,7, n = 64; asp1D333A, 90±43,3, n = 71. * p<0.0005 for asp1D333A vs. wild-type (Welch-test). (TIF) [file pgen.1004586.s017.tif]

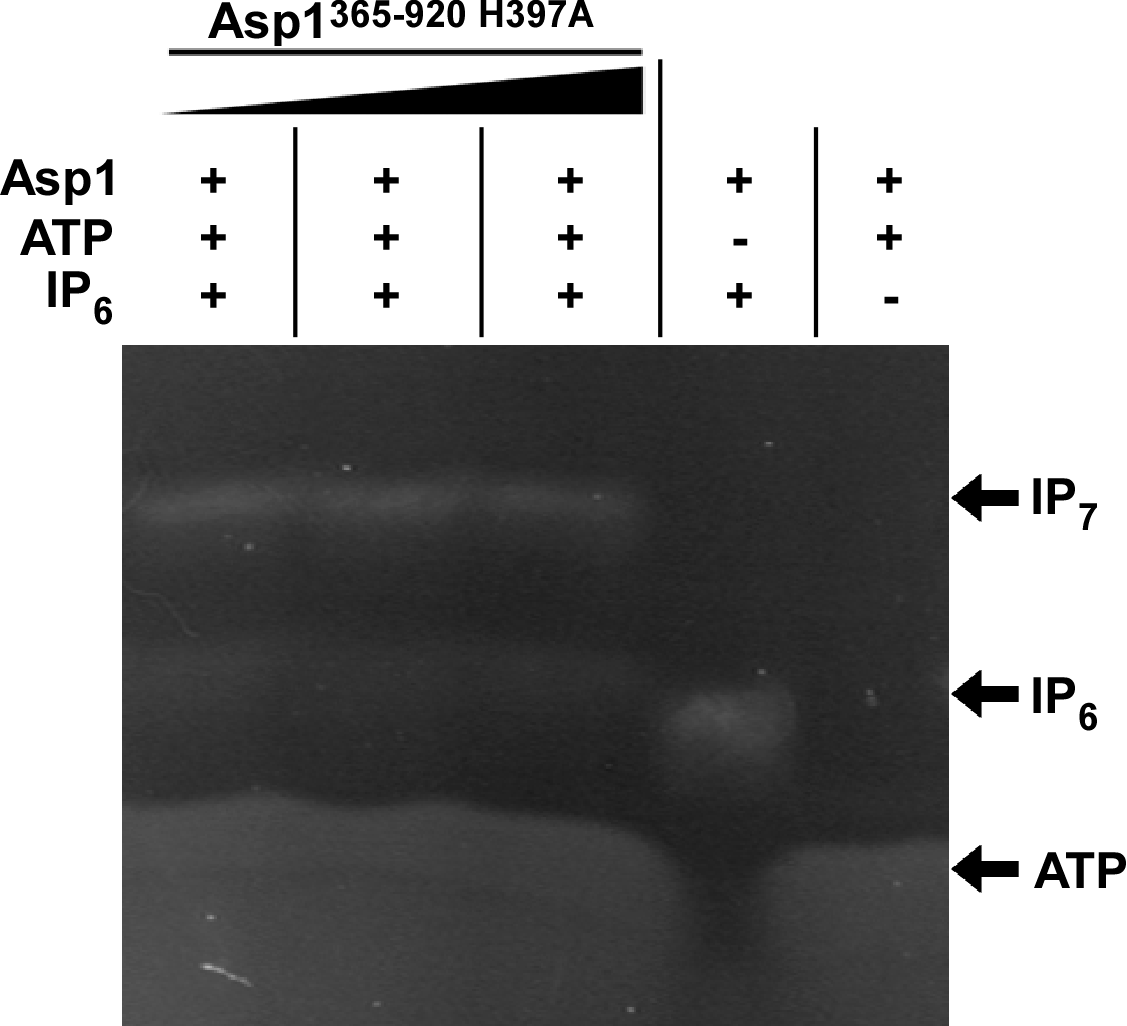

Supplement: Figure S18 — Generation of IP7 by GST-Asp1 with varying amounts (2,4,8 µg) of Asp1365-920 H397A. Enzymatic reaction was carried out as described in Figure 1C. −, component not present; +, component present. (TIF) [file pgen.1004586.s018.tif]
